# Identifying disease-specific genes based on their topological significance in protein networks

**DOI:** 10.1186/1752-0509-3-36

**Published:** 2009-03-23

**Authors:** Zoltán Dezső, Yuri Nikolsky, Tatiana Nikolskaya, Jeremy Miller, David Cherba, Craig Webb, Andrej Bugrim

**Affiliations:** 1 GeneGo Inc, Renaissance Drive, Saint Joseph, Michigan 49085, USA; 2 Program of Translational Medicine, Van Andel Research Institute, Bostwick Avenue NE, Grand Rapids, MI 49503, USA; 3 Vavilov Institute of General Genetics, Russian Academy of Sciences, Gubkina Str, Moscow, Russia

## Abstract

**Background:**

The identification of key target nodes within complex molecular networks remains a common objective in scientific research. The results of pathway analyses are usually sets of fairly complex networks or functional processes that are deemed relevant to the condition represented by the molecular profile. To be useful in a research or clinical laboratory, the results need to be translated to the level of testable hypotheses about individual genes and proteins within the condition of interest.

**Results:**

In this paper we describe novel computational methodology capable of predicting key regulatory genes and proteins in disease- and condition-specific biological networks. The algorithm builds shortest path network connecting condition-specific genes (e.g. differentially expressed genes) using global database of protein interactions from MetaCore. We evaluate the number of all paths traversing each node in the shortest path network in relation to the total number of paths going via the same node in the global network. Using these numbers and the relative size of the initial data set, we determine the statistical significance of the network connectivity provided through each node. We applied this method to gene expression data from psoriasis patients and identified many confirmed biological targets of psoriasis and suggested several new targets. Using predicted regulatory nodes we were able to reconstruct disease pathways that are in excellent agreement with the current knowledge on the pathogenesis of psoriasis.

**Conclusion:**

The systematic and automated approach described in this paper is readily applicable to uncovering high-quality therapeutic targets, and holds great promise for developing network-based combinational treatment strategies for a wide range of diseases.

## Background

While the utility of systems biology tools and approaches are increasing within scientific research, several fundamental challenges have limited their wide-spread adoption in both the basic and translational sciences. The identification of truly relevant networks that are causatively associated with the phenotype of interest is paramount to the field of systems biology [1-3]. Beyond the identification of an integrated network of interest, further analysis of the system is required to identify key target nodes that may represent novel therapeutic targets, or targets of the existing pharmacopeia. Beyond discovery, it is equally important to begin to consider how our expanding knowledge of molecular interactions may be translated into clinical applications in the area of molecular diagnostics and/or targeted therapeutics. Each of these areas requires the development of a more robust and systematic systems biology tool kit to permit the automated construction and further analysis of molecular networks. The publicly available molecular datasets anchored to well-characterized disease phenotypes provide an excellent framework upon which such methods can be developed and further validated.

In the typical pathway analysis, the first step is the identification of a characteristic molecule set from experimental data (e.g., differentially expressed genes associated with disease of interest). The association of experimentally identified genes and/or proteins with available pathway and protein interaction data provides the foundation for further analysis. This can be accomplished with the help of integrated commercial solutions such as MetaCore™ (GeneGo, Inc., St. Joseph, MI) [4], IPA (Ingenuity Systems, Mountain View, CA) or by combining publicly available tools such as Cytoscape [5] with internal or public protein interaction and pathway databases. The reconstruction of condition-specific networks is often based on the fact that biological networks are highly modular. In general, network modules refer to a group of directly connected or closely located (in terms of network distance) proteins in the global network that work together to achieve a distinct biological function [6,7].

Different variations of the shortest-path algorithm often serve to extract network modules, ultimately aiming to uncover the most-affected biological processes in condition-specific pathways. The algorithms are found either as built-in network reconstruction tools within commercial software packages or as open-source plug-in modules for Cytoscape. However, one fundamental issue facing this approach is the high interconnectivity seen in the biological networks due to the presence of a small number of hubs, that is, network nodes with hundreds or even thousands of connections [8]. In many cases, the shortest path between two nodes will logically traverse such hub(s). Although some of these connections may be biologically meaningful, many are artifacts arising from the presence of hubs in the network. Further analysis of network topology and graph statistics is needed to find pathways that are truly significant for a given molecular profile.

Several attempts have been made to address the artifacts generated by the small number of hubs in the biological network. Croes et al. [9], for example, proposed to weight nodes in metabolic networks based on their connectivity, giving a penalty to highly connected metabolites. Their results showed significant improvement in the accuracy of predicting known metabolic pathways: the authors reported up to 93.7% accuracy for predicting known reaction steps in pathways between pairs of metabolites. Another approach, implemented in MetaCore™, uses well-established canonical pathways as shortcuts while generating shortest paths in protein signaling networks. The algorithm gives preference to known signaling routes while reconstructing condition-specific networks. Recent work by Yu et. al [10] shifts the emphasis from high-degree hubs to nodes that are "bottlenecks" in the network, i.e., nodes that have a disproportional number of shortest paths going through them.

These approaches have potentially severe limitations. Always penalizing hubs might exclude them in situations where they play a truly important role in a condition-specific network. By the same token, always giving preference to known pathways limits the ability to generate new hypotheses in important signaling cascades. One approach to address this problem would be to consider the network topology in the context of a particular dataset (for example, a set of genes differentially expressed in a disease), and to evaluate the statistical significance of all the shortest paths within the framework of the global connectivity map.

Another fundamental issue in pathway analysis is how to use the results in guiding further laboratory research and clinical applications. The results of pathway analyses are usually sets of fairly complex networks or functional processes that are deemed relevant to the condition represented by the molecular profile. To be useful in a research or clinical laboratory, the results need to be translated to the level of testable hypotheses about individual genes and proteins within the condition of interest. Systems biology has become a key component in drug and biomarker discovery, to identify both the causative targets in disease networks in addition to potential biomarkers of target disruption. In such areas, systems biology is often used to create a hypothesis about a specific condition, and identify a small number of molecules that can be further interrogated in the laboratory for clear-cut answers to confirm or refute the hypothesis.

In this work, we present a method and associated computational algorithm which addresses the fundamental issues described above. Our method scores nodes in a network built from a set of experimentally derived, condition-specific genomic or proteomic profiles. The scoring is based on the role the nodes play in providing connectivity among genes or proteins of interest relative to their role in the global network. The method is therefore neutral with respect to the node's degree or centrality, i.e., the role of nodes with a high degree of physical connections is neutralized. Scores for truly significant nodes are enhanced, while the scores of those that appear in the networks by chance are reduced. In addition, the output of our algorithm is a set of prioritized network nodes along with their possible regulatory effects on other genes and proteins. This output provides the researcher with a series of scored and testable hypotheses associating individual components of the identified molecular network(s) with the phenotype of interest.

## Results

Consistent with other systems biology methodologies, the algorithm described here starts with a set of experimentally identified network nodes. In this study, we used previously published data from a microarray study of psoriatic lesions [11]. In this work gene expression was assayed in four patients with psoriasis using HUG95A Affymetrix microarray platform. The skin samples were taken from the same area of skin before and after the lesions receded; this kept any variation in gene expression profiles not related to the disease to a minimum. A t-test of gene expression was performed to compare lesions and healthy skin. Two hundred sixty-six genes were identified by the authors as being differentially expressed in psoriasis (p < 0.05, Additional file 1). This set of genes was mapped onto the global database of protein-protein interactions available in GeneGo's MetaCore™ platform. This database is a commercially available resource containing over 200,000 protein-protein and protein-small molecule interactions manually extracted from the literature by a group of experts. The method described can be readily applied to any of the publicly available protein-protein interaction databases such as BIND [12], DIP [13], HPRD [14] and IntAct [15].

To address the issue of network hubs providing most of the shortest path connectivity in biological networks, we assessed the relative contribution of every node in a condition-specific network relative to its role in the global network (see "Methods" section for algorithm description). Thus, the hubs which do not have any special role related to the set of differentially expressed genes were penalized, even though they may be highly connected. On the other hand, nodes that are truly relevant for providing connectivity among experimentally derived genes were highly scored regardless of the total number of interactions they have. We refer to this procedure as "topological significance scoring" because its results depend on the topology of protein network and define significance of a node with respect to providing network connectivity among the genes or proteins of interest.

### Initial validation with a set of publicly available data on psoriasis

We used 266 differentially expressed genes identified by t-test statistics (p < 0.05) in [11] as the input set and applied the algorithm described in the Methods section. In the algorithm, all network nodes corresponding to genes from the input list are considered "sources" and "targets", the source node being the starting point and the target node being the endpoint of directed shortest paths connecting differentially expressed genes among themselves. The rest of the nodes that the shortest path consists of are the "internal" nodes necessary to connect the source to the target node. The resulting condition-specific shortest path network (CSSPN) contained 3,652 internal nodes (Additional file 2). Each of these nodes was assigned a p-value according to the procedure described in the Methods section. One hundred forty-five of the differentially expressed genes from the input set were also internal to the network and thus were assigned p-values (Additional file 3). The rest of the differentially expressed genes were only present as sources or targets of directed pathways.

To evaluate whether or not the nodes deemed significant by our method are disease-related, we performed an automated search of abstracts in PubMed to find co-occurrence of the gene or protein name and the word "psoriasis" for all the proteins and genes that corresponded to nodes in the shortest path network (total of 3,652 nodes). It is understood that only some papers in which the names co-occur describe a functional relationship between the protein/gene and disease. However, it is reasonable to assume that the proportion of papers describing actual gene-disease relationships is independent of the node topological significance assigned by our method. Thus, co-occurrence frequency can serve as a relative measure of the algorithm's performance.

**Figure 1:**
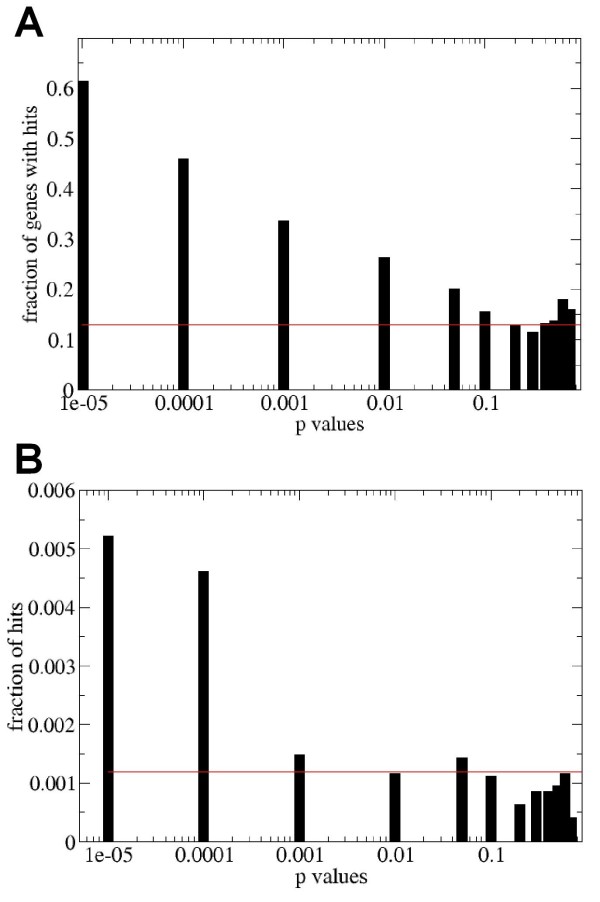
**(A) Fraction of genes with at least one co-occurrence with the word "psoriasis" in PubMed**. Genes corresponding to network nodes having small p-values show a significantly higher chance to co-occur with the word "psoriasis" than a random set of genes (red line). (B) Co-occurrence of a gene with the word "psoriasis", as a fraction of total number of PubMed hits for that gene. By normalizing by total number of hits per gene, we demonstrate that genes with small p-values indeed show significantly higher relevance to the disease.

In the first test, we pooled nodes into "significance bins" according to the order of magnitude of p-values our algorithm assigned. We then evaluated the fraction of all nodes in each bin which are associated with psoriasis by literature hits. The results shown in Figure 1a indicate that as many as 60% of the highest-scored nodes (p < 1e-05) co-occur in the literature with psoriasis. This compares with a mean of only 12% among the nodes whose p-value is > 0.1. This provides a good initial indication that our method can identify disease-related network nodes.

Next, we needed to verify that we are selecting genes specifically related to psoriasis rather than being generally well-studied genes that would have a high publication rate related to any disease context. Figure 1b shows the number of papers in which the gene's name co-occurs with the word "psoriasis" as a fraction of total number of papers mentioning that gene. Again, we see significant enrichment among highly scored nodes relative to the rest of the nodes in the set. To verify if the high-scored nodes are specifically related to psoriasis, we performed the search on the same set of genes for other diseases. We searched the number of papers in which the same genes and the world 'glaucoma' or 'cancer' co-occurs. As we expected, we did not find a positive correlation between the high scoring nodes and the number of literature hits (see Additional file 4 for glaucoma). We also tested our algorithm on another autoimmune disease, multiple sclerosis. The scaling of the pubmed hits was not as good as it was in the case of "psoriasis", but for low p values the fraction of hits was still slightly higher. This is expected because both diseases are autoimmune and therefore they probably share some of the affected pathways (Additional file 4). Collectively, these data confirm that the algorithm assigns high scores to psoriasis-related genes using this publically available dataset. Importantly, many of these high-scored network nodes are not differentially expressed in the microarray data set, but were inferred by the algorithm from the differentially expressed gene pattern and the analysis of network connectivity.

**Figure 2:**
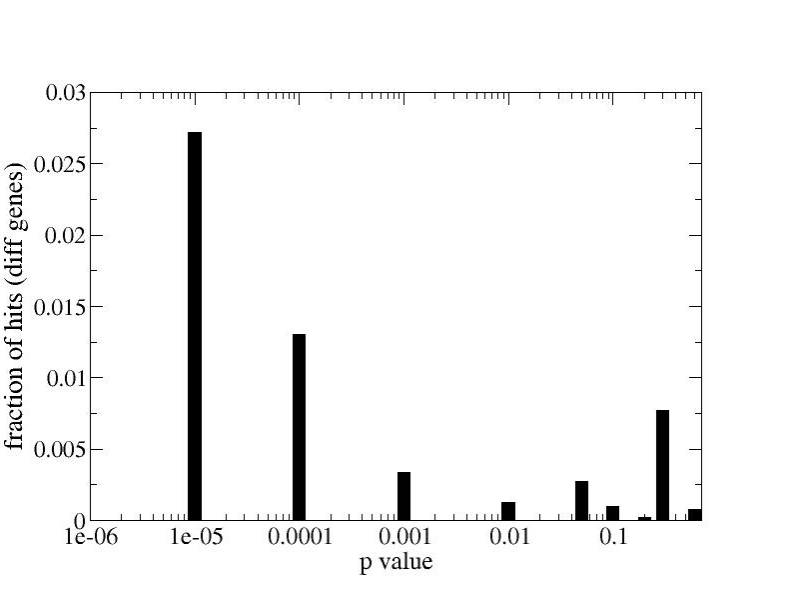
**Co-occurrence with the word "psoriasis" for differentially expressed genes as a fraction of total number of PubMed hits**. Due to the high interconnectivity of biological networks, many differentially expressed genes are also part of the shortest path network and were therefore assigned a p-value. Genes that are both differentially expressed and scored highly for network topology show the most relevance and specificity to psoriasis. Figure 1b shows that results presented here are not simply due to the fact that genes with small p-values are also the best studied.

As described above, approximately half of the genes differentially expressed in association with the psoriasis phenotype were also identified within the shortest paths network and therefore assigned p-value scores based on topological significance. To further explore this subset of genes, we generated a plot similar to that in Figure 1, showing the number of papers in which the name of a gene and disease co-occur as a fraction of all papers written about that gene (Figure 2). In this case the statistics are restricted only to differentially expressed genes. Genes with small p-values have a substantially higher fraction of hits with "psoriasis" than differentially expressed genes on average. The fraction of papers with "psoriasis" hits for all differentially expressed genes is on average 0.008, while for those with p-values < 1e-05, the fraction reaches 0.028. Thus, using the method of literature-based confirmation, the network topology-based algorithm is able to effectively prioritize differentially expressed genes identified from analysis of this single psoriasis data set.

### Functional validation of the protein prioritization algorithm

Based upon the results above, it would appear that highly scored network nodes are more likely to be associated with the disease phenotype relative to all other nodes in the shortest path network and differentially expressed nodes. To investigate these results further, we conducted a thorough functional study of the highly scored nodes from our analysis of psoriasis gene expression using the standard MetaCore™ software suite. We loaded two sets of genes into the software. The first set contained 266 differentially expressed genes identified by the t-test. The second set contained genes identified as topologically significant members of the shortest path network connecting differentially expressed genes. Genes were selected from the 3,652 genes by applying false discovery rate (FDR) filtering by rank-order of their topological p-values. A total of 202 genes passed FDR filtering using the experiment significance level of 0.001 (Additional file 5). First, we computed the global functional distribution of the data, in which each dataset was mapped onto the MetaCore™ collection of functional pathway maps (510 maps representing major functional blocks in cell signaling and metabolism). These maps are freely available . Enrichment statistics were calculated for these two data sets in the 510 cell signaling and metabolic function blocks in MetaCore™ assuming a hypergeometric distribution. The false discovery rate filter with an experiment significance level of 0.01 was applied to account for the fact that we are testing the significance of over 500 maps and the result may contain some false positives. Top scoring maps with detailed description were included in Additional file 4.

**Figure 3:**
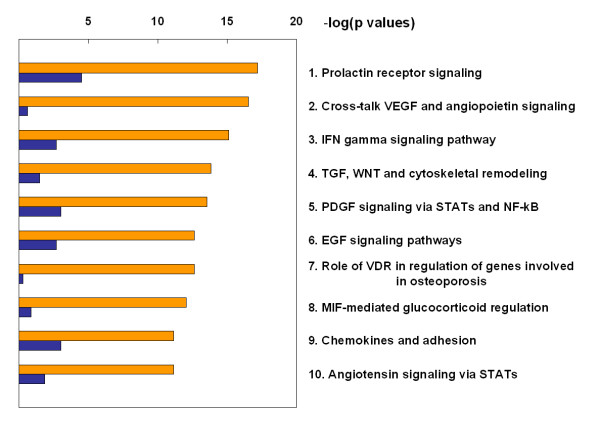
**Enrichment of pathway maps in differentially expressed (blue bars) and high-scored (orange bars) genes**. Many inflammatory processes are enriched in high-scored genes, in good agreement with known close relation between psoriasis and inflammation. Distributions are computed using MetaCore™.

To visualize the significance level of individual maps, a histogram of -log(p-value) is plotted showing each dataset separately (Figure 3). Orange bars represent enrichment of pathways for genes highly scored by our algorithm; blue bars represent enrichment in differentially expressed genes. Table 1 shows significant pathways for the combined dataset constructed as a union of topologically significant and differentially expressed genes. It is clear that statistically significant pathway maps have high relevance to inflammation, cell cycle, and cell adhesion processes. These results are in very good agreement with known facts regarding the role of these processes in the pathogenesis of psoriasis. The analysis of topologically high scoring nodes for down regulated genes in psoriasis resulted in similar conclusions for both the PubMed hits and functional analysis (Additional file 4).

**Table 1:** Top scoring maps for a combined set of differentially expressed genes and topologically significant proteins.

**Map**	**Map Folders**	**Cell process**	**p-value**	**Genes**
IFN gamma signaling pathway	Immune response	cytokine and chemokine mediated signaling pathway, immune response	1.88E-26	32/63
Prolactin receptor signaling	Growth factors	intracellular receptor-mediated signaling pathway, response to hormone stimulus	4.57E-24	30/62
Regulation of G1/S transition (part 2)	Cell cycle control	cell cycle	3.30E-22	22/33
Chemokines and adhesion	Cell adhesion	cytokine and chemokine mediated signaling pathway, cell adhesion	6.46E-22	45/174
EGF signaling pathway	Epidermal cell differentiation	intracellular receptor-mediated signaling pathway, response to extracellular stimulus	4.89E-21	28/64
PDGF signaling via STATs and NF-kB	Growth and differentiation	intracellular receptor-mediated signaling pathway, response to extracellular stimulus	5.18E-21	23/40
IGF-RI signaling	Growth and differentiation	intracellular receptor-mediated signaling pathway, response to extracellular stimulus	1.63E-20	29/72
AKT signaling	Function groups/Kinases	protein kinase cascade	5.96E-19	25/57
TGF, WNT and cytoskeletal remodeling	Cell adhesion	cell adhesion	6.15E-19	45/204

In the "Genes" column the first figure is the number of genes from the combined dataset on each map, the second figure is the total number of genes represented on each map.

The top-scored map for the combined dataset is the IFN-gamma signaling pathway (Table 1). A high level of saturation of this pathway with topologically significant nodes is in excellent agreement with the current views on the pathogenesis of psoriasis. The function of the inflammatory processes that induce the migration of interferon gamma-producing Th1 lymphocytes into the skin is thought to be central to the development of psoriasis. These Th1 lymphocytes are responsible for the pathologic reactions in psoriatic skin leading to keratinocyte hyperproliferation, small vessel proliferation, and neutrophilic infiltration [16]. Interestingly, the second top-scoring map is prolactin signaling. Even though the role of prolactin in psoriasis is not completely clear [17,18], recent studies suggest that it may enhance IFN-gamma-induced CXCL9, CXCL10, and CXCL11 production in keratinocytes via activation of STAT1, NF-kappaB, and IRF-1 through the JAK2 and MEK/ERK pathways and thus may promote type 1 T-cell infiltration into psoriatic lesions [19].

**Figure 4:**
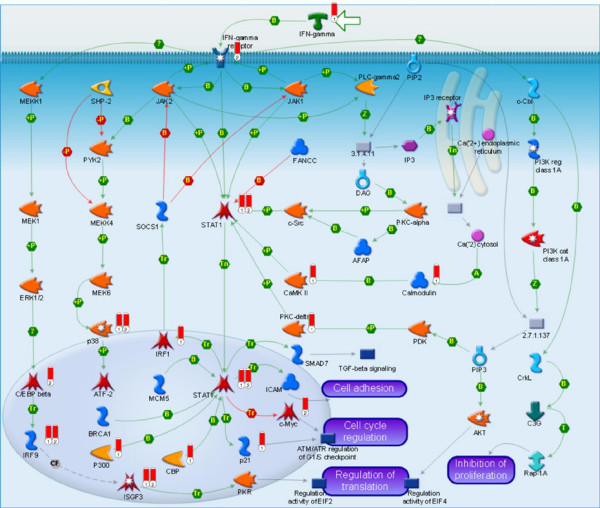
**IFN-gamma signaling pathway**. Mapped on the pathway are the set of high-scored network nodes (set #1) and the set of differentially expressed genes (set #2). The datasets are visualized by thermometer-like symbols next to protein icons. Differentially expressed genes with a well-established relation to disease (e.g. STAT1, c-Myc) are cross-validated by high statistical scoring. Many additional important elements of the pathway are identified by topological scoring – IFN-gamma, IRF-1, PKC-delta, CaMK II, CBP.

Further mechanistic investigation of the maps reveals that combining differentially expressed genes and topologically significant nodes helps to reconstruct a more complete picture of disease-related pathways, with topological scoring indentifying key elements of the pathways that are missed by gene expression profiling. Figure 4 shows the top-scoring IFN-gamma signaling pathway map. Individual sets of data are represented by small thermometer-like icons next to proteins. The set of topologically scored proteins is labeled #1 and the set of differentially expressed genes is #2. Two trends are visible on this map. First, some key genes known to be related to psoriasis are cross-validated by both high topological scores and differential expression (two thermometers are present next to these proteins). The examples include STAT1, an important element in transducing IFN-gamma signals, and p38, the key regulator of apoptosis and stress response. Both of these molecules are highly implicated in signaling related to the disease [19]. Second, many important elements of the pathway are identified by topological scoring alone. These include IFN-gamma which triggers this pathway as well as IRF-1, PKC-delta, CaMK II, CBP and other important modulators of this pathway's activity. Even though these molecules were not identified as differentially expressed, their selection by the topological scoring algorithm allows reconstruction of a much more complete picture of this key pathway. While IFN-gamma [20], IRF-1 [19,21] and CBP [22,23] were already associated with psoriasis in independent studies, there is no direct evidence in the literature regarding involvement of PKC-delta and CaMKII in the disease. On the other hand, some studies indicate that other PKC isozimes [24] and calcium signaling in general [25] may play role in the pathogenesis of psoriasis. Generally, molecules such as protein kinases are unlikely to change their expression levels in a way that is detectable by current microarray techniques. Nonetheless, such molecules often play key regulatory roles in disease-affected pathways. Topological scoring identifies them due to their central position in signaling cascades connecting differentially expressed genes. The proposed algorithm is capable of performing a dual task: cross validating disease target genes initially identified by gene expression and suggesting a pivotal role for new targets that could not be inferred from gene expression profiling alone.

### Functional hubs and "drugable" network modules

When our algorithm is applied, a network node can be assigned multiple p-values, each relating it to one of the differentially expressed genes (See Methods section for details). The p-value can be viewed as the strength of a "functional link" between the node in the shortest path and the differentially expressed gene. Strong functional link (small p-value assigned by our method) implies that the node in the shortest path plays a key role in providing connectivity between the gene and the rest of the differentially expressed set. Some nodes may have many functional links with good strength. We call such nodes "functional hubs" (see Methods).

We investigated topologically significant nodes and functional hubs which resulted from the analysis of psoriasis gene expression data in the context of known treatments for this disease. As the first step, all nodes with high topological scores were screened against the database of targets for drugs currently on the market or molecules in the development pipeline and were ranked based on their "drugability". From the set of 202 topologically significant genes for psoriasis, we identified 97 proteins which are drug targets based on the drug content of MetaCore™ (Additional file 6). We also identified 20 out of the 97 proteins as functional hubs (see Methods). A literature search of the top five proteins from the topologically significant nodes revealed that four of them were targets for drugs documented to have a positive effect on psoriasis: chondroitin sulfate [26], retinoic acid [27], mycophenolate mofetil [28], and alclometasone dipropionate [29]. Some drugs had multiple targets from our list. For example, retinoic acid targets were enriched in the list of topologically significant proteins. Indeed, 20% of the top 20 proteins and 25% of the functional hubs were targets for retinoic acid, which is higher than the 10% of the set of differentially expressed genes being targets for the same drug. This is in good agreement with the fact that retinoic acid is a widely used treatment in dermatology, including psoriasis [27]. Furthermore, although 50% of the functional hubs were targets for at least one of the above-mentioned drugs (compared with 10% for the differential genes), there was only *one *functional hub identified as also being differentially expressed, Interferon regulatory factor 9. These results serve as an additional validation of our technique, showing that it is able to identify many known psoriasis drug targets while indicating that functional hubs could be used to predict likely new targets.

To gain further insight into functional hubs and their relevance to psoriasis, we built network modules consisting of sets of genes linked to hubs by upstream or downstream functional connections. The genes corresponding to these modules were mapped onto standard Gene Ontology and other process and disease ontologies available in MetaCore™ [30]. This allowed us to prioritize functional hubs based on their relevance to psoriasis-related processes or psoriasis-related genes. We obtained 18 functional hubs and corresponding network modules that were enriched in genes related to skin diseases and conditions (for enrichment analysis of modules, see Additional file 7). Out of 11 modules for divergence hubs, 5 were enriched in skin diseases, while out of 20 modules for convergence hubs, 13 were enriched in skin diseases. Notably, this analysis allowed identification of 5 potential novel targets, i.e., proteins that are functional hubs with relevance to skin disease, but for which no drug information was found. These are ROR-alpha, NFKBIA, Thombospondin 1, KNG, and NF-kB1 (highlighted by yellow in Additional file 7).

**Figure 5:**
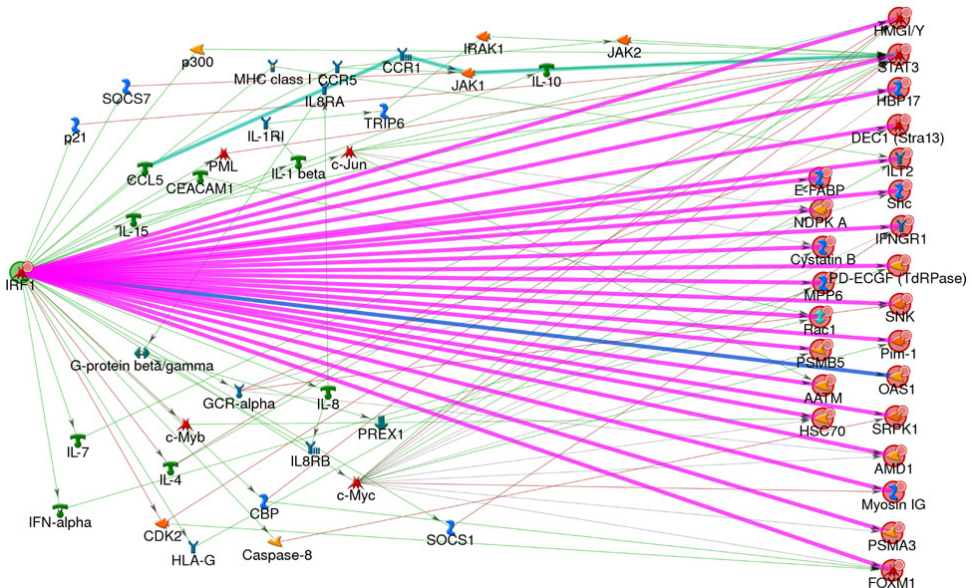
**IRF-1 functional disease network reconstructed from psoriasis expression profile**. The network was generated from psoriasis data as described in the "Results" section. IRF-1(left) was identified as a "divergence" hub functionally linked to multiple downstream target genes differentially expressed in psoriasis. Functional links are highlighted in pink. In MetaCore we have also reconstructed possible physical pathways underlying functional influence (thin lines). The network has 75% of its nodes associated with skin diseases by annotations in MetaCore.

Thus, the end-result of our analysis of a disease gene expression profile is a set of network modules (originating from functional hubs) prioritized based on their potential functional impact and drug availability. For each module, the analysis identifies affected biological processes and disease-related genes. We call these sub-networks *drugable network modules*. An example of such a module is generated from interferon regulatory factor 1 (IRF-1), which is identified as one of the "divergence" hubs functionally linked to multiple downstream targets (Figure 5). Functional links were imported into MetaCore™ using MetaLink™, a parser for interaction data. The network was analyzed with respect to the disease-related genes (as recorded in MetaCore™), and the result shows that it has high relevance to skin diseases, with about 75% of its nodes associated with this disease category (for enrichment of all modules, see Additional file 7).

## Discussion

In this study we have introduced a novel procedure for assigning topological significance scores to nodes in protein interaction networks. The scoring is based on node's role in providing the shortest paths connectivity among genes or proteins from experimentally-derived set. There are several advantages of this method which make it a powerful tool in the analysis of high-throughput molecular data such as gene expression or proteomics profiles. First, our method generates significance scores based on the input of a particular molecular profile, such as a set of differentially expressed genes. Thus it finds nodes that are significant with respect to a condition or a disease represented by such profile. This can be contrasted with general topological properties of network nodes such as clustering coefficient, centrality and degree which characterize nodes independently of any condition-specific molecular data. Second, it assigns scores to all nodes participating in the shortest path network, whether or not they are part of the input experimental set. Therefore it allows identification of many important elements of signal transduction pathways which themselves do not come up as differentially expressed genes or proteins.

In this study we have tested the ability of our approach to identify network nodes associated with a selected disease (psoriasis) using the input of empirically defined differentially expressed genes, and to predict which network nodes are key mediators of the disease phenotype through the analysis of network topology. As an indication of success, nodes which are assigned high significance (small p-values) by our algorithm are also several times more likely to be related to psoriasis in the literature, than nodes with low significance. The result shows that our approach can be applied as a "sorting" method for prioritizing gene and protein sets from high throughput assays. For example, many microarray studies yield hundreds of differentially expressed genes. Pursuing further research on all of them is prohibitively expensive. High topological scores assigned to some of these genes could serve as additional evidence, helping to select most promising candidates for detailed investigation. Topological scoring could also point to several new candidates – genes with exceptionally high topological scores that do not display differential expression. For example, our analysis assigned high significance scores to several protein kinases and transcription factors (IRF-1, CaMKII, PKC, etc.) which are not differentially expressed themselves but whose activity was inferred based on significant downstream effects reflected in the gene expression profile. As shown above, some of these proteins (IRF-1, PKC-beta) were already linked to psoriasis in other independent studies, while others (CaMKII, PKC-delta) may represent novel targets.

Mechanistic investigation of highly scored network nodes shows that many of them play key roles in pathways for inflammation, cell cycle, and cell adhesion – all highly associated with the pathogenesis of psoriasis. These pathways are highly enriched in topologically significant nodes overall. This indicates that topologically significant nodes can be used for identifying molecular mechanisms of the disease or condition of interest. When pathway enrichment analysis is performed for both differentially expressed genes and topologically significant nodes the results are dominated by maps strongly enriched in topologically significant nodes (Figure 3). This is due to the fact that topologically significant nodes tend to represent key regulatory elements of signaling cascades, while differentially expressed genes are more likely to be found among target genes whose expression is regulated by these cascades. By design, expertly compiled pathway maps tend to focus on signaling cascades – bearers of core functionality. Target genes which in many cases could be regulated by multiple signaling pathways tend not to be concentrated on any particular map. Moreover, many key regulators have hundreds of target genes and not nearly all of them are represented on pathway maps. Hence the enrichment analysis of topologically significant nodes reveals signaling pathways containing proteins that have high relevance to regulation of differentially expressed target genes as a group, even though target genes themselves are not well represented on maps for these pathways.

In addition to topological scoring, our method assigns "functional links" between differentially expressed genes and nodes in the shortest paths. Nodes with multiple functional links represent "functional hubs". Functional hubs are condition-specific: their functional links are determined by topological scoring procedure and defined in the context of a particular condition-specific molecular profile. Many functional hubs themselves do not have large numbers of direct physical interactions with other proteins. Rather they provide unique "bridges" between multiple differentially expressed genes. Some functional hubs may have the majority of their links downstream while others have them upstream. The first type of hub is essentially "in control" of many functionally significant connections, while the second type is "being controlled" by a multitude of such connections. Thus we suggest that these "divergence" and "convergence" hubs may play fundamentally different roles in the disease and drug response, and affecting them with a drug may have different consequences. We consider functional hubs to be priority drug targets, because of their key positions in pathways regulating multiple genes whose expression is affected in disease. Since special role of functional hubs is defined with respect to a disease-specific dataset, it is quite possible that at least some of them play just a minimal role in signaling pathways under normal conditions. Thus, unlike nodes with many physical interactions, targeting functional hubs with drugs is more likely to maximize impact on the disease while minimizing effects on general physiological processes.

Our analysis also suggests that network modules centered on functional hubs are likely to be relevant to the disease phenotype, in this case psoriasis. Importantly, such modules can be readily tested in the lab by affecting corresponding hubs by drugs or other methods, such as RNA interference. This opens new possibilities in systematically predicting and validating drug targets by using the concept of drugable network modules.

## Conclusion

We developed a novel statistical approach for scoring network nodes in the protein interaction network for their relevance to a disease phenotype or other condition of interest. This scoring is accomplished through a combined analysis of high throughput molecular assays and topology of the protein network. We applied our method to gene expression profiles from psoriasis patients and identified many known and several new drug targets for psoriasis and biological pathways in which they participate. These findings were in excellent agreement with the current knowledge on the pathogenesis of psoriasis. Finally, we introduced the concept of functional hubs and corresponding "drugable" network modules. Our analysis suggests that these modules are the most promising candidates to be targeted by drugs in order to maximize the impact on the disease. The results indicate that our approach opens new possibilities for systematic identification of the new promising drug targets and prioritizing them for further detailed investigation.

## Methods

### Algorithm for node prioritization and reconstructing significant pathway modules

#### Topological scoring of nodes

**Figure 6:**
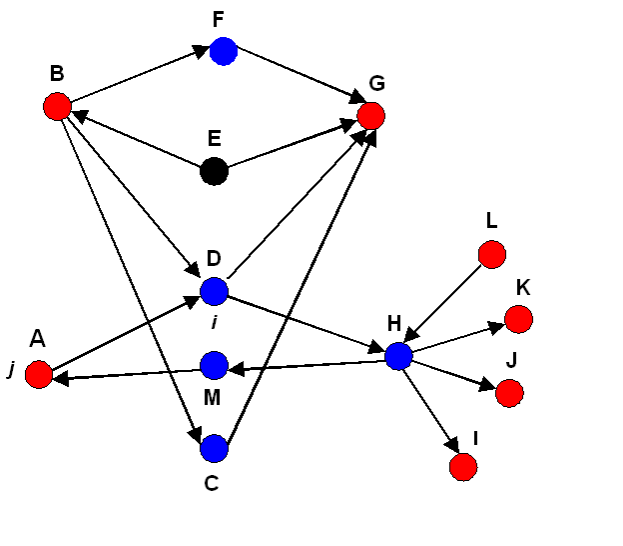
**Set of experimentally derived nodes *K *is colored red**. We connect them by shortest path network *S *(blue nodes). The rest of the global network is represented by black nodes. In this example, the size of the global network *N *= 13, *K *= 7, and *S *= 5. The number of possible shortest path networks between node *B *and each of the other nodes in the global network which can contain D is 11 (*N*-2). The number of such networks which contain node *D *is 7 (*N*_*BD *_= 7). On the other hand, the number of shortest path networks containing *D*, among those connecting only nodes from the set *K*, is 5 (*K*_*BD *_= 5). The significance (p-value) for node *D *with respect to node *B *and set *K *can be calculated as *p*_*BD *_= *p*(*N*-2, *N*_*BD*_, *K*-1, *K*_*BD*_). Similarly, we can calculate the other p-values for *D *with respect to *A, G*, *K*, *J*, *I*, and *L*, and then pick the smallest value and assign it as the significance of node *D *in the sub-network defined by the nodes of interest (red nodes). The nodes can be classified as internal (*F*, *D*, *C*, *H*, and *M*), source (*A*, *B*, and *L*) and target (*A*, *G*, *K*, *J*, and *I*) nodes.

Our algorithm starts with a set of experimentally identified network nodes. To understand how this algorithm works, let's assume that *K *is a set of experimentally derived nodes of interest (e.g., nodes representing differentially expressed genes). *K *is a subset of a global network of size *N*. The first step is the construction of a directed shortest path network connecting each node in *K *to other nodes in *K*, traversing via other nodes in the global network. If there are multiple shortest paths of equal length between two nodes in K, then all of the nodes from the multiple paths are included in the shortest path network for that pair, *S*, which is a subset of *N *and contains nodes in addition to *K*. Some nodes from *K *may become "internal" in *S*, that is, they are lying on the shortest paths, while the rest are either "source" or "target" terminals of the shortest paths (Figure 6). All nodes in *S *that are not in *K *are by definition "internal" nodes. For future reference, we call *S *a condition-specific shortest path network (CSSPN). The building of this shortest path network is executed by a modified version of the standard breadth-first search described elsewhere (for details, see for example [31]).

Let us consider node *i *∈ *S*, an internal node, and *j *∈ *K*, one of the nodes of the experimental set. In addition to *S*, we calculate the shortest paths between *j *and every other node (except *i*) in the global network, wherever such shortest paths exist (up to *N*-2 pairs). Then we count how many of these node pairs have node *i *present in at least one of the shortest paths; this number is *N*_*ij *_≤ *N*-*2*. On the other hand, we count how many times node *i *occurs in at least one shortest path of node pairs when connecting *j *to all other nodes in *K*. This number is *K*_*ij *_≤ *K*_*j *_≤ *K*-*1 *(we assume node *i *is not differentially expressed; otherwise *K*_*j *_≤ *K*-*2*). Note that we count node *i *only once for every pair from *K*, even though it may be part of multiple linear shortest paths connecting the same pair.

Under the "null" hypothesis, node *i *has no special role in connecting node *j *to the rest of differentially expressed genes in *K*. Thus, the probability of finding *i *in the shortest paths connecting *K*_*ij *_or a larger number of node pairs originating or terminating at node *j *follows a cumulative hypergeometric distribution. This problem can be recast as selection without replacement. *N*_*ij *_node pairs containing *i *as an internal node in the shortest paths connecting *j *to all other nodes in the global network can be considered as a set of "marked" node pairs. On the other hand, a set of *K*-*1 *pairs consisting of the node *j *and the rest of *K*-*1 *experimentally derived nodes represent a "selection". If node *i *has no special role for connecting *j *to the rest of the nodes in *K*, then the number of marked shortest path networks in the selection should follow the hypergeometric distribution



where *p*_*ij*_(*K*_*ij*_) is the probability of finding node *i *in the shortest paths connecting *K*_*ij *_number of node pairs in the differentially expressed set among those originating or terminating at node *j*. The p-value is calculated as the cumulative distribution of the function above. We repeat this procedure for all nodes in *K*, calculating up to *K *p-values for each node *i *in the network of shortest paths connecting differentially expressed genes. Each of these p-values shows relevance of node *i *to individual members of the set *K*. As we want to identify the nodes that are statistically significant to at least one or more members of the experimental set, we define the "topological significance" score associated with node *i *as the minimum of the *p*_*ij *_values. We note that our method, unlike betweenness centrality does not count the actual number of shortest paths between the pairs of nodes, but rather it counts the number of instances a node is part of the shortest path network between the node pairs. More importantly, our technique considers fractions of differentially and non-differentially expressed genes connected by shortest paths containing the node that is being evaluated. In this context it is not concerned with the paths bypassing the node of interest. In contrast, the betweenness centrality measure is based on relative numbers of shortest paths going via the node of interest and those bypassing it.

Upon request the authors will provide the topological scoring algorithm code and a publicly accessible hyperlink for a web-based version of the algorithm.

#### Directionality and functional hubs

For any node *j*, the shortest paths connecting it to other nodes could be divided into two categories: those leading from node *j *to other nodes and those leading to node *j *from other nodes. From the perspective of an internal node *i *connecting *j *to other nodes in *K*, these will be *"incoming" *and *"outgoing" *paths, respectively. In the calculations described above, we bundled these two sets of paths into a single number, *K*_*ij*_, representing overall connectivity. On the other hand, we can also consider them separately. The procedure for this is similar to the one described above. Thus, we end up with two p-values, *p*_*in *_and *p*_*out*_, respectively. The p-value score can be viewed as the strength of the incoming or outgoing "functional link" between the internal node and the differentially expressed gene. The lower the p-value, the stronger functional link is. In the psoriasis study, nodes having at least 10 incoming or outgoing functional links below the p-value threshold of 10^-4 ^were considered convergence or divergence "functional hubs", respectively.

## Authors' contributions

AB designed the study and wrote the manuscript, ZD performed statistical data analysis, contributed to the conception and design of the algorithm and the writing of the manuscript, YN and TN contributed to functional analysis of the data, JM, DC and CW contributed to the conception and design of the study and helped writing the manuscript. All authors read and approved the final manuscript.

## Supplementary Material

Additional file 1 **List of differentially expressed genes in psoriasis. **Two hundred sixty-six genes were identified as being differentially expressed in psoriasis by comparing gene expression of psoriatic and healthy skin (p < 0.05, t-test). Click here for file

Additional file 2 **Topological scoring of genes**. The set of differentially expressed genes in psoriasis was used as an input for the topological scoring algorithm. All nodes (3,652) from the shortest path network connecting the differentially expressed genes were assigned p-values. Click here for file

Additional file 3 **Topological scoring of differentially expressed genes.** One hundred forty-five differentially expressed genes were part of the shortest path network and were assigned p values. Click here for file

Additional file 4 **Top scoring maps and analysis of pub-med hits with other diseases. **Images and detailed description of top scoring maps from the enrichment analysis of the combined set of differentially expressed and topologically significant genes. The file also includes the functional analysis of down-regulated genes in psoriasis and pub-med hit statistics for glaucoma and multiple sclerosis. Click here for file

Additional file 5 **Topologically significant genes.** Topologically significant genes were selected from the shortest path nodes by applying false discovery rate. A total of 202 genes passed FDR filtering using the experiment significance level of 0.001. Click here for file

Additional file 6 **Drug targets and functional hubs. **From the set of 202 topologically significant genes, we identified 97 drug targets and 20 functional hubs. Click here for file

Additional file 7 **Enrichment analysis of functional hubs.** We built network modules consisting of sets of genes linked to functional hubs by upstream or downstream functional connections. Functional enrichment analysis was performed to prioritize hubs based on their relevance to psoriasis-related processes or psoriasis-related genes. Click here for file

## References

[B1] Chuang HY, Lee E, Liu YT, Lee D, Ideker T (2007). Network-based classification of breast cancer metastasis. Mol Syst Biol.

[B2] Calvano SE, Wenzhong X, Richards DR, Felciano RM, Baker HV, Cho RJ, Chen RO, Brownstein BH, Cobb JP, Tschoeke SK, Miller-Graziano C, Moldawer LL, Mindrinos MN, Davis RW, Tompkins RG, Stephen, Lowry SF (2005). A network-based analysis of systemic inflammation in humans. Nature.

[B3] Vert JP, Kanehisa (2003). Extracting active pathways from gene expression data. Bioinformatics.

[B4] Bugrim A, Nikolskaya T, Nikolsky Y (2004). Early prediction of drug metabolism and toxicity: systems biology approach and modeling. Drug Discov Today.

[B5] Shannon P, Markiel A, Ozier O, Baliga NS, Wang JT, Ramage D, Amin N, Schwikowski B, Ideker T (2003). General transcription factor specified global gene regulation in archaea. Genome Res.

[B6] Hartwell LH, Hopfield JJ, Leibler S, Murray AW (1999). From molecular to modular cell biology. Nature.

[B7] Alon U (2003). The Tinkerer as an Engineer. Science.

[B8] Barabasi A-L, Oltvai ZN (2004). Understanding the Cell's Functional Organization. Nature Reviews Genetics.

[B9] Croes D, Couche F, Wodak SJ, van Helden J (2006). Inferring Meaningful Pathways in Weighted Metabolic Networks. J Mol Biol.

[B10] Yu H, Kim PM, Sprecher E, Trifinov V, Gerstein (2007). The Importance of Bottlenecks in Protein Networks: Correlation with Gene Essentiality and Expression Dynamics. PLoS Comput Biol.

[B11] Kulski JK, Kenworthy W, Bellgard M, Taplin R, Okamoto K, Oka A, Mabuchi T, Ozawa A, Tamiya G, Inoko H (2005). Gene expression profiling of Japanese psoriatic skin reveals an increased activity in molecular stress and immune response signals. J Mol Med.

[B12] Bader G, Betel D, Hogue CW (2003). BIND: the biomolecule interaction network database. Nucleic Acids Res.

[B13] Xenarios I, Salwinski L, Duan XJ, Higney P, Kin SM, Eisenberg D (2002). DIP, the database of interacting proteins: a research tool for studying cellular networks of protein interactions. Nucleic Acids Res.

[B14] Peri S, Navarro JD, Amanchy R, Kristiansen TZ, Jonnalagadda CK, Surendranath V, Niranjan V, Muthusamy B, Gandhi TKB, Gronborg M, Ibarrola N, Deshpande N, Shanker K, Shivashankar HN, Rashmi BP, Ramya MA, Zhao Z, Chandrika KN, Padma N, Harsha HC, Yatish AJ, Kavitha MP, Menezes M, Choudhury DR, Suresh S, Ghosh N, Saravana R, Chandran S, Krishna S, Joy M, Anand SK, Madavan V, Joseph A, Wong GW, Schiemann WP, Constantinescu SN, Huang L, Khosravi-Far R, Steen H, Tewari M, Ghaffari S, Blobe GC, Dang CV, Garcia JG, Pevsner J, Jensen ON, Roepstorff P, Deshpande KS, Chinnaiyan AM, Hamosh A, Chakravarti A, Pandey A (2003). Development of human protein reference database as an initial platform for approaching systems biology in humans. Genome Res.

[B15] Hermjakob H, Montecchi-Palazzi L, Lewington C, Mudali S, Kerrien S, Orchard S, Vingron M, Roechert B, Roepstorff P, Valencia A, Margalit H, Armstrong J, Bairoch A, Cesareni G, Sherman D, Apweiler R (2004). IntAct – an open source molecular interaction database. Nucleic Acids Res.

[B16] Ghoreschi K, Mrowietz U, Rocken M (2003). A molecule solves psoriasis? Systemic therapies for psoriasis inducing interleukin 4 and Th2 responses. J Mol Med.

[B17] Chuang E, Molitch ME (2007). Prolactin and autoimmune diseases in humans. Acta Biomed.

[B18] Giasuddin AS, El-Sherif AI, El-Ojali SI (1998). Prolactin: does it have a role in the pathogenesis of psoriasis?. Dermatology.

[B19] Kanda N, Watanabe S (2007). Prolactin Enhances Interferon-γ-Induced Production of CXC Ligand 9 (CXCL9), CXCL10, and CXCL11 in Human Keratinocytes. Endocrinology.

[B20] Yao Y, Richman L, Morehouse C, de los Reyes M, Higgs BW, Boutrin A, White B, Coyle A, Krueger J, Kiener PA, Jallal B (2008). Type I interferon: potential therapeutic target for psoriasis?. PLoS ONE.

[B21] Odanagi M, Kikuchi Y, Yamazaki T, Kaneko T, Nakano H, Tamai K, Uitto J, Hanada K (2004). Transcriptional regulation of the 230-kDa bullous pemphigoid antigen gene expression by interferon regulatory factor 1 and interferon regulatory factor 2 in normal human epidermal keratinocytes. Exp Dermatol.

[B22] Westergaard M, Henningsen J, Johansen C, Rasmussen S, Svendsen ML, Jensen UBSchrøder HD, Staels B, Iversen L, Bolund L, Kragballe K, Kristiansen K (2003). Expression and localization of peroxisome proliferator-activated receptors and nuclear factor kappaB in normal and lesional psoriatic skin. J Invest Dermatol.

[B23] Wang YN, Chang WC (2003). Induction of disease-associated keratin 16 gene expression by epidermal growth factor is regulated through cooperation of transcription factors Sp1 and c-Jun. J Biol Chem.

[B24] Gönczi M, Papp H, Biro T, Kovacs L, Csernoch L (2002). Effect of protein kinase C on transmembrane calcium fluxes in HaCaT keratinocytes. Exp Dermatol.

[B25] McKenzie RC, Oda Y, Szepietowski JC, Behne MJ, Mauro T (2003). Defective cyclic guanosine monophosphate-gated calcium channels and the pathogenesis of psoriasis. Acta Derm Venereol.

[B26] Verges J, Montell E, Herrero M, Perna C, Cuevas J, Perez M, Moller I (2005). Clinical and histopathological improvement of psoriasis with oral chondroitin sulfate: a serendipitous finding. Dermatol Online J.

[B27] Weindl G, Roeder A, Schafer-Korting M, Schaller M, Korting HC (2006). Receptor-selective retinoids for psoriasis: focus on tazarotene. Am J Clin Dermatol.

[B28] Zwerner J, Fiorentino (2007). Mycophenolate mofetil. Dermatol Ther.

[B29] Aggerwal A, Maddin S (1982). Alclometasone dipropionate in psoriasis: a clinical study. J Int Med Res.

[B30] Gene Ontology Consortium (2006). The Gene Ontology (GO) project in 2006. Nucleic Acids Res.

[B31] Newman MEJ (2001). Scientific collaboration networks. II. Shortest paths, weighted networks, and centrality. Phys Rev E.

