# Supplementary material for: Identifying disease-specific genes based on their topological significance in protein networks
Source: BMC Syst Biol. 2009 Mar 23;3:36. doi: 10.1186/1752-0509-3-36 (PMC2678983; doi:10.1186/1752-0509-3-36)
Supplement: Additional file 4 — Top scoring maps and analysis of pub-med hits with other diseases. Images and detailed description of top scoring maps from the enrichment analysis of the combined set of differentially expressed and topologically significant genes. The file also includes the functional analysis of down-regulated genes in psoriasis and pub-med hit statistics for glaucoma and multiple sclerosis. [file 1752-0509-3-36-S4.pdf]

I. **Top scoring maps**

1.IFN gamma signaling

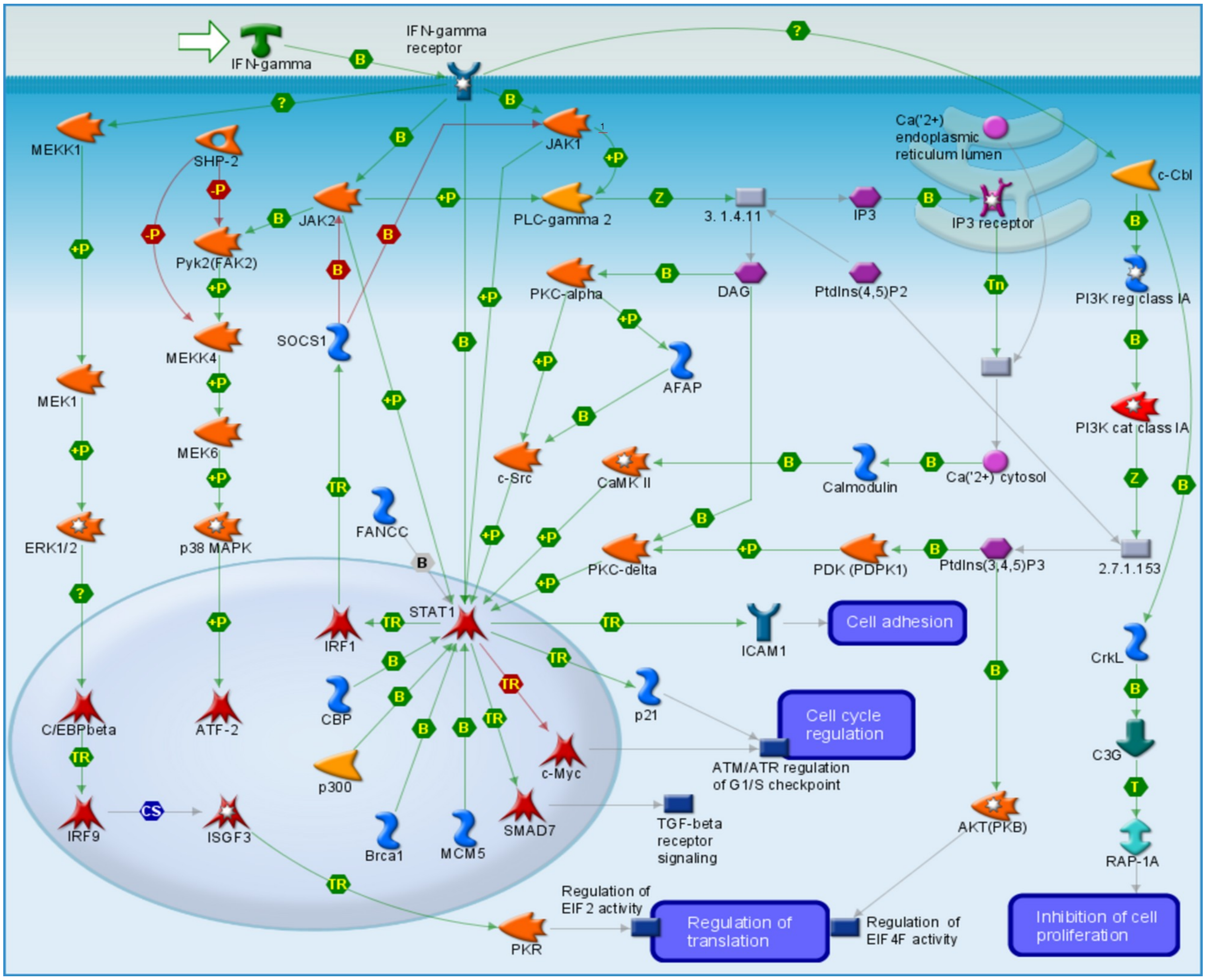

Interferon-gamma signaling

Interferons (IFNs) are pleiotropic cytokines that mediate anti-viral responses, inhibit proliferation and participate in immune surveillance and tumor suppression by inducing the transcription of a number of IFN-stimulated genes. The IFN family includes two main classes of related cytokines, type I IFNs and type II IFN. There are many type I IFNs: interferon-alpha, interferon-beta and many others. By contrast, there is only one type II IFN, interferon-gamma (**IFN-gamma**) that is produced by activated T cells and natural killer (NK) cells. **IFN-gamma** exerts its effects on cells by interacting with the specific **IFN-gamma receptor** that is composed of two subunits, IFNGR1 and IFNGR2. **IFN-gamma receptor** is expressed on surfaces of nearly all cells. Binding of **IFN-gamma** to its receptor induces oligomerization of the receptor and activation, via trans-phosphorylation, of the receptor-associated Janus kinases 1 and 2 (**JAK1** and **JAK2**). The activated JAKs phosphorylate the intracellular domain of the receptor (e.g., tyrosine 440 of human IFNGR1) that serves as a docking site for Signal transducer and activator of transcription 1 (**STAT1**) **. STAT1** is phosphorylated on tyrosine 701, undergoes dimerization, translocates to the nucleus and regulates gene expression by binding to gamma-activated

sequence (GAS) elements in the promoters of IFN-gamma-regulated genes. Some kinases can phosphorylate **STAT1** at serine 727 (Ser727). This phosphorylation is not required for **STAT1** translocation to the nucleus or for its binding to the promoters. However, it is essential for the full transcriptional activation. These kinases include Protein kinase C delta (**PKC-delta**) and Calcium/calmodulin-dependent protein kinase II (**CaMK II**).

Precise mechanisms of IFN-gamma-induced activation of these kinases are not clear. However, it was shown that **IFN-gamma** activates Phosphatidylinositol 3-kinase (PI3K)/ v-AKT murine thymoma viral oncogene homolog (**AKT**) signaling pathway, perhaps via the adapter Cas-Br-M ecotropic retroviral transforming sequence (**c-Cbl**) that binds regulatory subunit of PI3K **(PI3K reg class 1A). PKC-delta** is an effector of the PI3K pathway. Although the mechanism of PI3K-dependent **PKC-delta** activation is unclear, PI3K-dependent phosphorylation of **PKC-delta** by 3-Phosphoinositide dependent protein kinase-1 (**PDK (PDPK1)**) was demonstrated.

Also **IFN-gamma** induces **c-Cbl** mediated activation of v-CRK avian sarcoma virus CT10 oncogene homolog-like **(CrkL)**. This provides a link between the **IFN-gamma receptor** and the Rap guanine nucleotide exchange factor 1 (**C3G**) and results in the **IFN-gamma** -dependent activation of RAP1A, member of RAS oncogene family (**Rap1A**), a protein known to exhibit tumor suppressor activity and mediate growth inhibitory responses.

IFN-gamma also induces phosphorylation of Phospholipase C gamma 2 (**PLC-gamma 2**) by **JAK1/2**. Diacylglycerol (**DAG**) is the product of the enzymatic activity of the **PLC-gamma 2**. It can activate some of the protein kinase C isoforms of, including **PKC-alpha.** The **PKC-alpha** can stimulate tyrosine-protein kinase SRC-1 (**c-Src**) activity. Although **PKC-alpha** can phosphorylate the **c-Src** directly, Actin filament associated protein (**AFAP**) is essential for this **c-Src** activation. **c-Src** in its turn activates **STAT1** by phosphorylation on tyrosine 701. This IFN-gamma-induced **PLC-gamma 2**/ **PKC-alpha**/ **c-Src**/ **STAT1** pathway leads to the expression of Intercellular adhesion molecule 1 (**ICAM-1**) gene.

There are many known **STAT1** -targets in IFN-gamma-mediated signaling. These are SMAD family member 7 (**SMAD7**), Interferon regulatory factor 1 (**IRF1**) and proteins involved in cell cycle regulation, e.g., v-Myc myelocytomatosis viral oncogene homolog (**c-Myc**) and Cyclin-dependent kinase inhibitor 1A (**p21**).

IRF1 participates in the activation of the Suppressor of cytokine signaling-1 (**SOCS-1**). The **SOCS-1** protein is critical for inhibiting **IFN-gamma** responses. **IFN-gamma** induces expression of **SOCS1** indirectly, by inducing the expression of the **IRF-1** transcription factor via **STAT1**. **IRF-1** in turn stimulates transcription of the **SOCS-1** gene.

Several proteins interact with **STAT1** and modulate its transcriptional activity: CREB-binding proteins (**CBP** and **p300**), Minichromosome maintenance protein 5 (**MCM5**) and Breast cancer susceptibility gene 1 (**BRCA1**). **CBP** and **p300** possess histone acetyl transferase activity and function as co-activators. **MCM5** and **BRCA1** associate with phosphorylated **STAT1** and enhance its transcriptional activity.

In addition, **IFN-gamma** may activate JAK-STAT-independent pathways.

Calcium-dependent tyrosine kinase PTK2B protein tyrosine kinase 2 beta (**Pyk2(FAK2)**) is a substrate for **JAK2**. **Pyk2(FAK2)** phosphorylates Mitogen-activated protein kinase kinase kinase 4 (**MEKK4**). Phosphorylated **MEKK4** in turn phosphorylates Mitogen-activated protein kinase kinase 6 (**MEK6**). Subsequently, **MEK6** phosphorylates **p38 MAPK** that phosphorylates and activates Activating transcription factor 2 (**ATF-2**). Protein-tyrosine phosphatase 2C (**SHP-2**) regulates this signaling pathway by dephosphorylating **MEKK4** and its activating kinase, **Pyk2(FAK2)**.

Another pathway stimulated by **IFN-gamma** involves Mitogen-activated protein kinase kinase kinase 1 (**MEKK1**), Mitogen-activated protein kinase kinase 1 (**MEK1**) and Mitogen-activated protein kinases 1 and 3 (**ERK1/2**). **MEKK1**/ **MEK1**/ **ERK1/2** cascade regulates activity of CCAAT/enhancer binding protein beta (**C/EBP-beta**) and C/EBP-beta-driven expression of Interferon regulatory factor 9 (**IRF9**) gene. **IRF9** is a subunit of **ISGF3** transcription complex that participates in interferon signaling

2. Prolactin receptor signaling

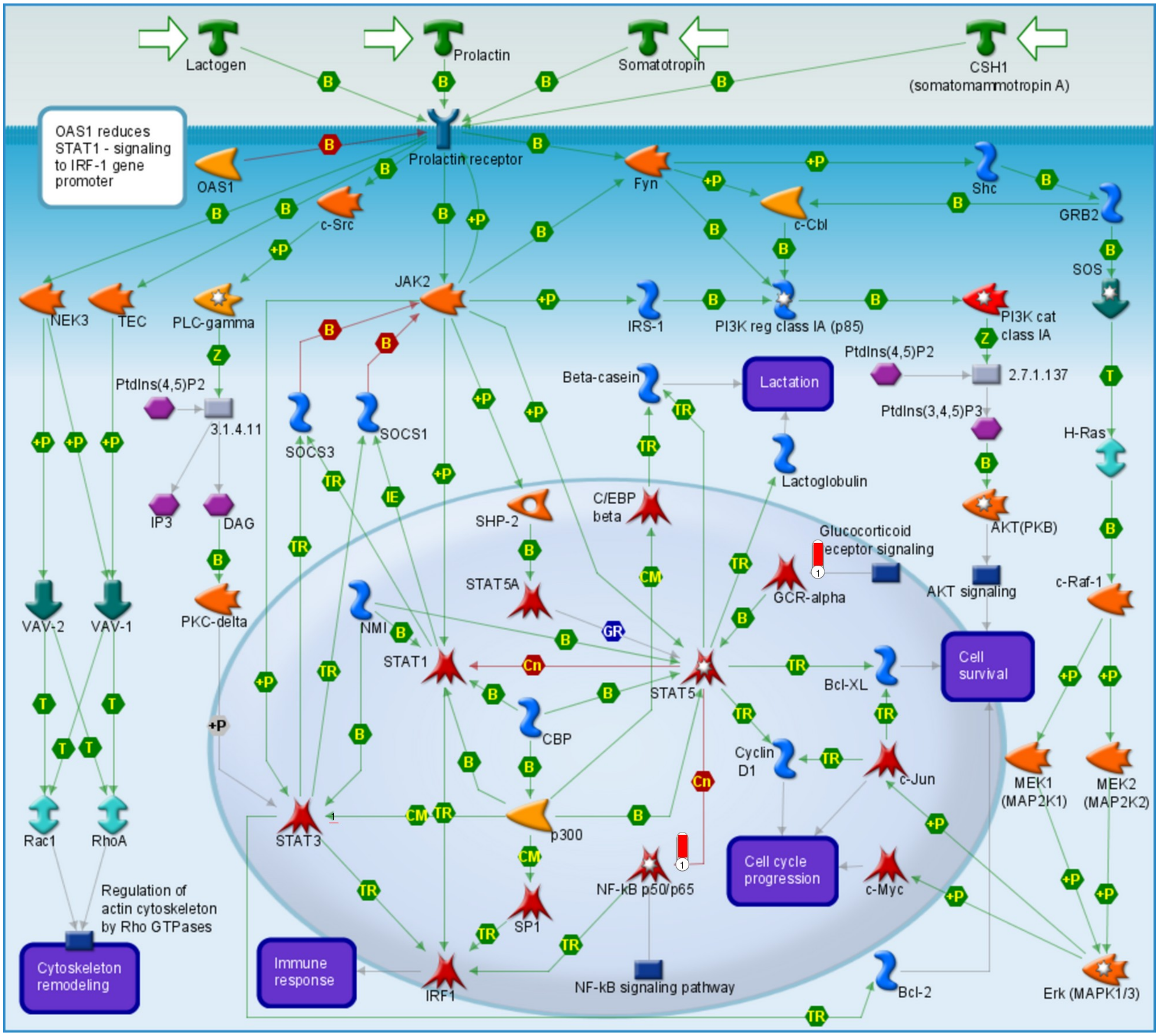

Prolactin is a polypeptide hormone secreted by the pituitary gland and to a lesser extent by numerous extrapituitary tissues. This hormone affects a great amount of physiological processes [1]. Numerous biological functions have been attributed to this hormone's activity, ranging from reproduction and

lactation to growth and development, from endocrinology and metabolism to brain and behavior, as well as immune regulation. **Prolactin** is a primary factor required for the growth and terminal differentiation of mammary epithelial cells as determined by the induction of transcription of milk protein genes required for lactation.

The initial step in **Prolactin** action is the binding to specific membrane cytokine receptor, **Prolactin receptor**. **Prolactin receptor** has an extracellular ligand-binding domain and intracellular domain. **Prolactin** is one of a family of related hormones including growth hormones **Somatotropin**, **Lactogen** and **CSH1 (somatomammotropin A)** that also bind to **Prolactin receptor**.

The cytoplasmic domain of the **Prolactin receptor** displays no enzymatic activity, but signals through activation of associated cytoplasmic tyrosine kinases, such as Janus kinase 2 **(JAK2**), V-src sarcoma viral oncogene homolog and FYN oncogene related to SRC FGR YES (**c-Src** and **Fyn**), NIMA-related kinase 3 (**NEK3**) and Tec protein tyrosine kinase (**TEC)**.

JAK2 activity stimulates **Prolactin receptor** dimerization and phosphorylation. Activated receptor through **JAK2** recruits Signal transducers and activators of transcription **(STAT**), in particular **STAT1**, **STAT3** and **STAT5** (**STAT5A** and **STAT5B**), and stimulates **STAT** s tyrosine phosphorylation. The phosphorylated **STAT** s dimmerize and translocate to the nucleus, resulting in the initiation of transcription of Interferon-regulatory factor-1 (**IRF-1**) and milk protein genes (such as **Beta-casein** and **Lactoglobulin**) in lymphocytes and mammary gland cells, respectively.

In the nucleus **STAT** s interact with coactivators **CBP** (CREB binding protein), **p300**, and N-myc interactor **(NMI**). **STAT5** transcriptional activation can be cooperatively enhanced by the alpha form of Nuclear receptor subfamily 3 group C member 1 (**GCR-alpha**) and CCAAT/Enhancer binding protein-beta **(C/EBPbeta**) to induce the transcription of **Beta-casein** gene. **Prolactin** stimulation of mammary cells leads to the nuclear translocation of Tyrosine phosphatase non-receptor type 11 (**SHP-2**) as a complex with **STAT5A** and binding of this complex to DNA, determining the milk protein gene transcription.

STAT5 factors also induce the transcription of **Cyclin D1** (which regulates cell cycle progression) and the antiapoptotic factor BCL2-like 1 (**Bcl-XL**).

In response to **Prolactin receptor** stimulation activated **STAT** s translocate into the nucleus and bind to the interferon-gamma activation sequence (GAS) in the promoter region of target genes. **STAT1** and **STAT3** have been shown to stimulate the transcription of the immediate early gene **IRF-1** in lymphocytes. **STAT1** activation of **IRF-1** promoter is enhanced by the constitutive factor Sp1 transcription factor (**SP1**), and coactivators E1A binding protein p300 (**p300**) and CREB binding protein (**CBP**).

In response to lymphocyte stimulation transcription factors **STAT1** and Nuclear factor kappa B (**NF-kB**) synergistically activate the **IRF-1** promoter, via the GAS and **NF-kB** elements, respectively. **STAT5B** has been demonstrated to inhibit the **IRF-1** transcription, and this inhibition is dependent upon **Prolactin receptor** stimulation. **STAT5B** inhibition does not require binding to the GAS element, but is mediated by squelching of limiting amounts of **p300**/ **CBP** coactivators necessary for gene transcription.

In addition, association of 2',5'-oligoadenylate synthetase (**OAS1**) with the **Prolactin receptor** inhibits **STAT1** signaling to the **IRF-1** promoter. Suppressors of Cytokine Signaling **(SOCS**) gene expression is mediated by **STAT3** and **STAT1**. **SOCS1** and **SOCS3** involve in negative regulation of **JAK2** and **STAT5** -dependent **Beta-casein** transcription. **Prolactin receptor** dimerization also induces the Mitogen-activated protein kinases pathway via **JAK2** and **Fyn** kinases activation. The complex formations of **Fyn**/ SHC transforming protein (**Shc**), **Shc**/ **GRB2**, and **Grb2**/Son of sevenless

homolog (**SOS**) induce **Shc**/ **GRB2**/ **SOS**/v-Ha-ras Harvey rat sarcoma viral oncogene homolog (**H-Ras**)/Mitogen-activated protein kinase kinase 1 and 2 (**MEK1** and **MEK2**)/Mitogen-activated protein kinase 3/1 (**ERK1/2**) cascade, ultimately activating Jun oncogene (**c-Jun**) and **c-Myc** transcription factors necessary for cell cycle progression.

Fyn and **JAK2** also activate Phosphatidylinositol-3 kinase (**PIK3**)/V-akt murine thymoma viral oncogene homolog 1 (**AKT(PKB)**)-pathway leading to cell survival. **Fyn** phosphorylates regulatory subunit of PIK3 (**PIK3 reg class 1A**). **JAK2** is required for the phosphorylation of insulin receptor substrate **IRS-1**. The role of **IRS-1** is to provide docking sites for **PIK3 reg class 1A** that activates catalytic subunit (**PIK3 cat class 1A**). Adaptor protein **c-Cbl**, which is phosphorylated by **Fyn**, in complex with **PIK3 reg class 1A** and **GRB2**, resulting in the activation of PI3K.

JAK2 also phosphorylates Phospholipase C gamma (**PLC-gamma**), activating Protein kinase C delta (**PKC-delta**) via Diacylglycerol (**DAG**). **PKC-delta** phosphorylates and activates **STAT3** downstream of **Prolactin receptor** signaling.

The **Prolactin receptor** dependent interactions of **NEK3** with VAV 1 and VAV2 guanine nucleotide exchange factors (**VAV1** and **VAV2**) and **Tec** with **VAV1** regulate cytoskeleton remodeling via activation of small GTPases (Ras homolog gene family member A (**RhoA**) and Ras-related C3 botulinum toxin substrate 1 (**Rac1**)).

3. Regulation of G1/S transition

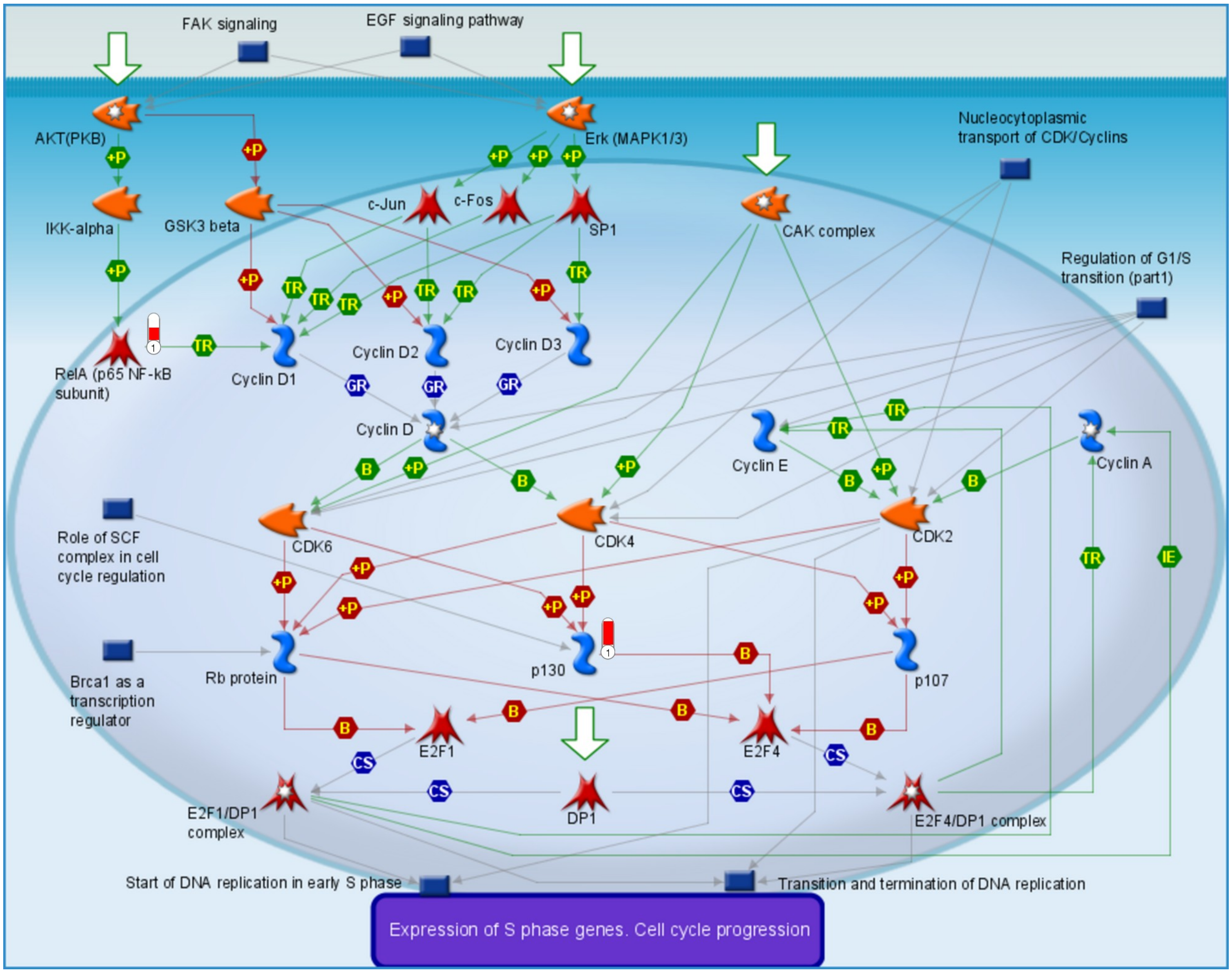

Regulation of G1/S transition (part 2)

The commencement of the cell cycle coincides with the production and the stabilization of the **Cyclin D**. The D-type cyclins are essential for synchronization of the cell cycle machinery with extracellular signals. Expression and stability of **Cyclin D** is monitored by growth factor receptors and focal adhesion-mediated signaling pathways.. Expression of **Cyclin D** may be activated through MAPK-cascade (via **SP1**, **c-Fos**, **c-Jun** transfactors) and/or through **AKT**/ **IKK**/ **NF-KB** pathway. In addition, **AKT** may inhibit **GSK3 beta**, thus preventing degradation of **Cyclin D** via the **GSK3** -dependent pathway.

Cyclins D are positive-regulatory partners of cyclin-dependent kinase 4 (**CDK4**) and cyclin-dependent kinase 6 (**CDK6**). Accumulating of **Cyclin D**/ **CDK** complexes is activated by phosphorylation of **CDK** s by **CAK complex**.

CDK s inhibit retinoblastoma tumor suppressors (pRB)-family proteins (**Rb protein**, **p107** and **p130**). pRB-family members are believed to function through their effects on the transcription of genes regulated by the **E2F** transcription factors. **CDK4** or **CDK6** phosphorylate pRB-family members, thereby liberating **E2Fs** (for example, **E2F1** or **E2F4**). These transcription factors associate with **DP1**

and together they induce expression of **Cyclin E**, **Cyclin A** and some other proteins necessary for DNA replication and the beginning of S phase. **Cyclin E** and **Cyclin A** are positive-regulatory partners of cyclin-dependent kinase 2 (**CDK2**). It is remarkable that both **Cyclin D**/ **CDK4** (or **CDK6**) and **Cyclin E/CDK2** are necessary for induction of expression of **Cyclin A**.

Activity of **CDK** s and **Cyclines** is inhibited by cell cycle kinase inhibitors (for example, **p27KIP** and other, see map). During transition from G1 phase to S phase, **p27KIP** is exposed to ubiquitin-mediated degradation by 26S proteasome.

4. Chemokines and adhesion

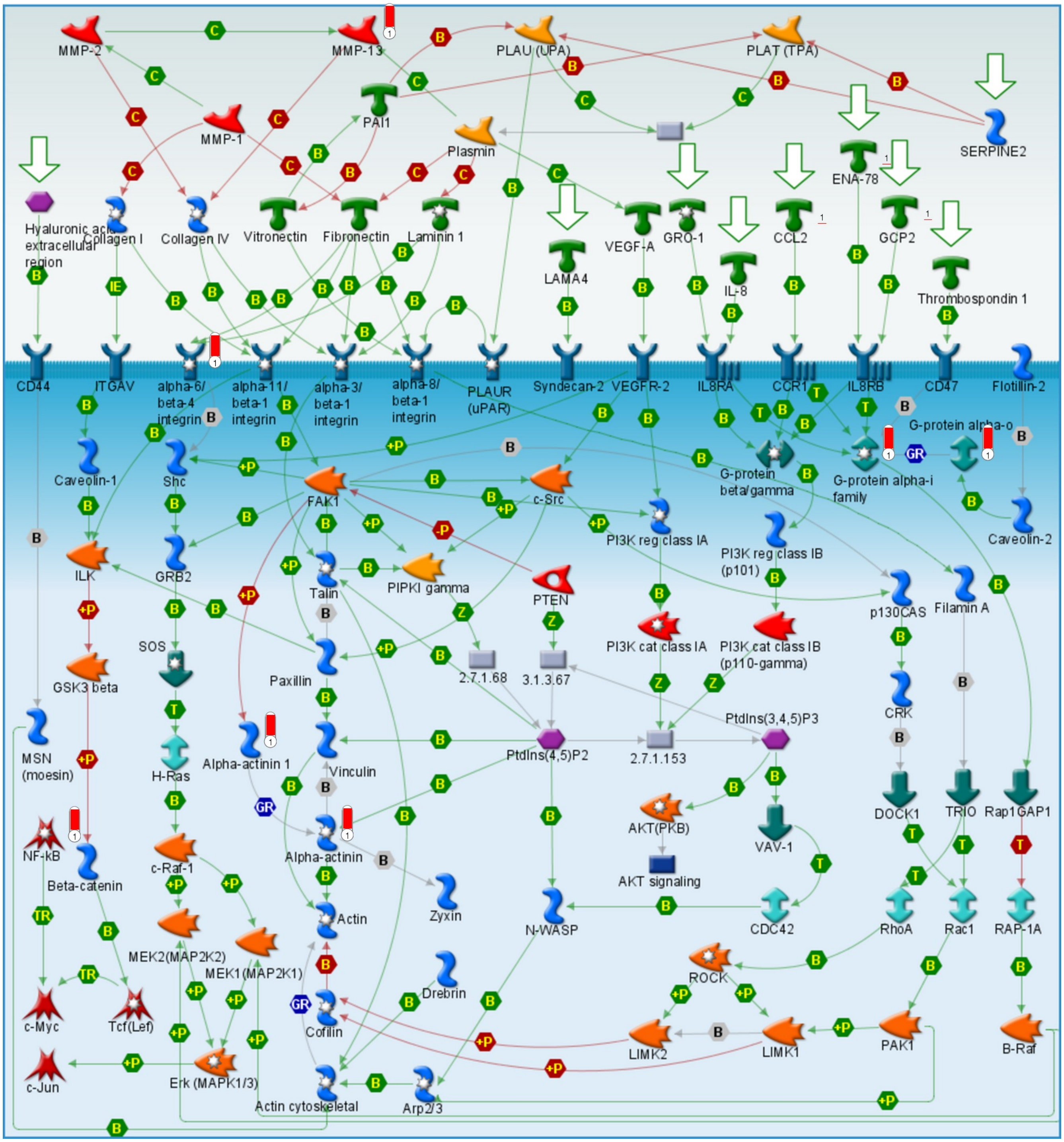

5. EGF signaling pathways

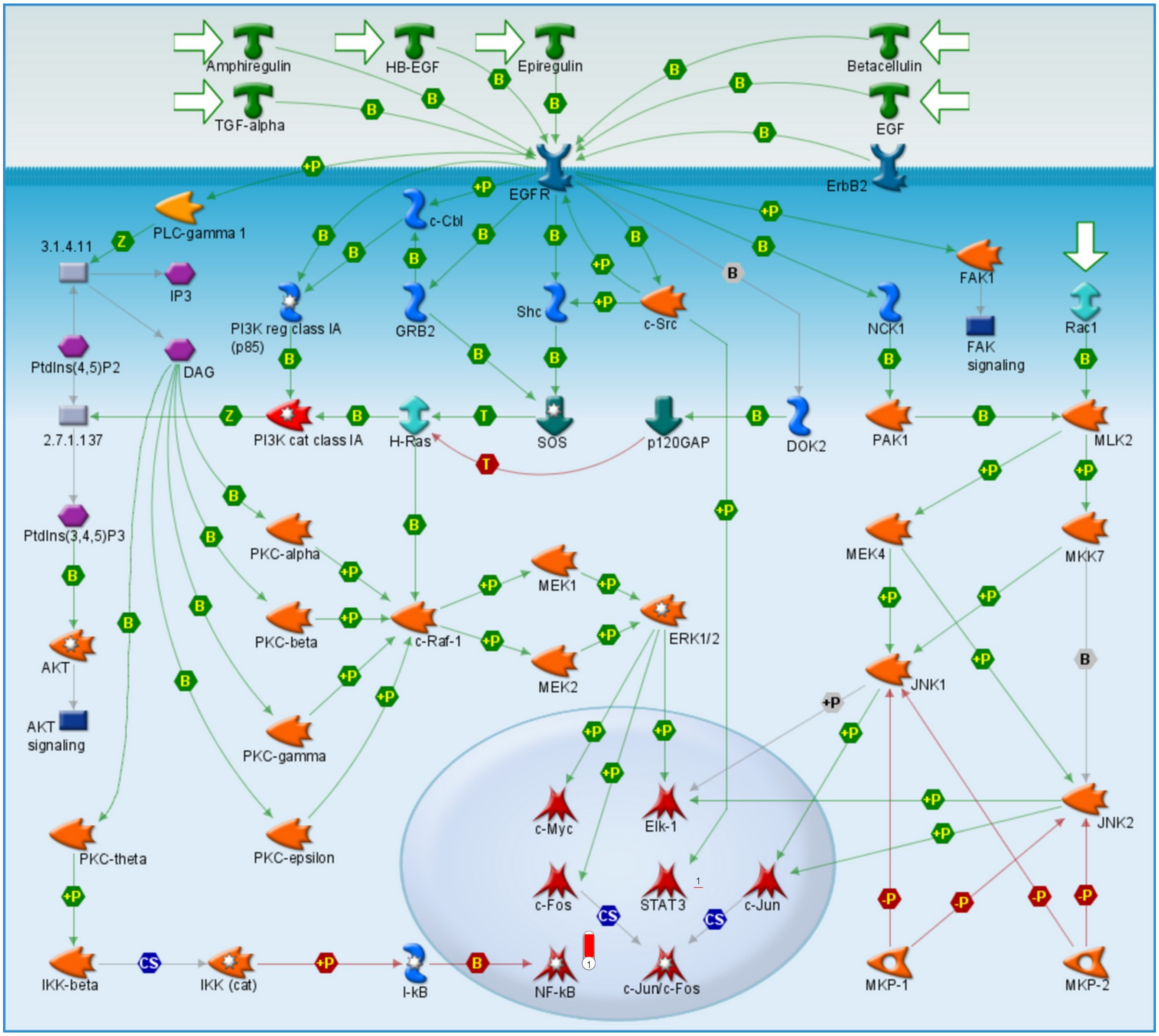

Epidermal growth factor receptor (**EGFR**) belongs to the **ERBB** family of receptor tyrosine kinases that contains four closely related members **EGFR** and ERBB2-4. They couple the binding of the extracellular growth factor ligands to intracellular signaling pathways that regulate diverse biologic responses, including proliferation, differentiation, cell motility, and survival.

Six ligands of **EGFR** are known. These are Epidermal growth factor (**EGF**), **Amphiregulin,** Transforming growth factor alpha (**TGF-alpha**), **Betacellulin**, Heparin binding EGF-like growth factor (**HB-EGF**), and **Epiregulin**.

ErbB2 is a unique member of the ERBB family in that it does not bind any of the known ligands with high affinity. However, it is the preferred heterodimeric partner for other **EGFRs**.

The ligand-induced receptor dimerization and subsequent autophosphorylation of distinct tyrosine residues creates docking sites for various membrane-targeted proteins. The cytoplasmic mediators that

bind to **EGFR** phosphotyrosine residues are either the adaptor proteins, such as SHC transforming protein 1 (**Shc**) **,** Growth factor receptor-bound protein 2 (**GRB2**) **,** Cas-Br-M ecotropic retroviral transforming sequence (**c-Cbl**) **,** Docking protein 2 (**DOK2**) and NCK adaptor protein 1 (**NCK1**), or enzymes, such as Phospholipase C gamma 1 (**PLC-gamma 1**), v-Src sarcoma viral oncogene homolog (**c-Src**) and PTK2 protein tyrosine kinase 2 (**FAK1**).

The adaptors **Shc** and **GRB2** recruit the exchange factor Son of sevenless homolog 1 (**SOS**) and form the complex consisting of **Shc**, **GRB2** and **SOS**. Activated **SOS** activates small GTPase v-Ha-ras Harvey rat sarcoma viral oncogene homolog (**H-RAS**) by its conversion from the inactive GDP-bounding state to the active GTP-bounding state. The activated **H-RAS** stimulates v-Raf-1 murine leukemia viral oncogene homolog 1 (**c-Raf-1)**/ Mitogen-activated protein kinase kinase 1 and 2 (**MEK1 and MEK2**)/ Mitogen-activated protein kinase 1 and 3 (**ERK1/2**) kinase cascade that leads to activation of the transcription factors ELK1 member of ETS oncogene family (**Elk-1**), v-Myc myelocytomatosis viral oncogene homolog (**c-Myc**), and v-Fos FBJ murine osteosarcoma viral oncogene homolog (**c-Fos**).

The adaptor **DOK2** associates with the GTPase-activating protein RAS p21 protein activator 1 (**p120GAP**) that reinforces intrinsic GTPase activity of **H-RAS,** thereby inactivating **H-RAS**. As a result, **DOK2** can attenuate activation of the EGF-stimulated mitogen-activated protein kinase (MAPK) cascade.

The adaptor **NCK1** couples **EGFR** stimulation to the activation of another MAPK-cascade, the JNK kinase cascade. **NCK1** recruits p21-Activated kinase 1 (**PAK1**). **NCK1**/ **PAK1** complex binds Mitogen-activated protein kinase kinase kinase 10 (**MLK2**) and activates the JNK cascade consisting of **MLK2/** Mitogen-activated protein kinase kinase 4 and 7 (**MEK4** and **MKK7**) **/** Mitogen-activated protein kinase 8 and 9 (**JNK1** and **JNK2**). The recruitment of the cascade to the activated membrane receptor localizes **MLK2** on the plasma membrane where it is activated by its known upstream effectors, such as Ras-related C3 botulinum toxin substrate 1 (**Rac1**). Stimulation of JNK cascade results in activation of the transcription factors **Elk-1,** Jun oncogene (**c-Jun**) and some others. Dual specificity phosphatases 1 and 4 (**MKP-1** and **MKP-2**) attenuate activation of the JNK cascade.

The adaptor **GRB2** also binds via its SH3 domain with proline-rich regions of the **c-Cbl** protein. **c-Cbl** is tyrosine-phosphorylated by tyrosine kinase upon stimulation via the EGF receptor. EGF stimulation induces the association of **c-Cbl** with the regulatory p85 subunit of the Phosphatidylinositol 3-kinase (**PI3K reg class IA (p85)**).

Activated **PI3K cat class IA** converts Phosphatidylinositol 4,5-biphosphate (PtdIns(4,5)P2) to Phosphatidylinositol 3,4,5-triphosphate (**PtdIns(3,4,5)P3**). The latter is a second messenger involved in regulation of various processes. **PtdIns(3,4,5)P3** associates with the inner surface of the plasma membrane and promotes the recruitment of proteins with pleckstrin homology (PH) domains. One of such proteins is serine/threonine kinase v-AKT murine thymoma viral oncogene homolog (**AKT**). It is the essential mediator of various cell processes, such as apoptosis, cell cycle, protein synthesis, regulation of metabolism.

Enzymes such as **PLC-gamma 1** or the cytoplasmic tyrosine kinase **c-Src** tie **EGFR** activation to the generation of secondary messengers and calcium metabolism or to mitogenic signaling cascades, respectively. **EGFR** recruits and phosphorylates **PLC-gamma 1**.

Phosphorylated **PLC-gamma 1** generates Diacylglycerol (**DAG**) and Inositol-1,4,5-trisphosphate (**IP3**) from PtdIns(4,5)P2.

DAG activates many isoforms of protein kinase C (PKC), including conventional isoforms alpha, beta, and gamma (**PKC-alpha, PKC-beta, and PKC-gamma**), as well as **PKC-epsilon** and **PKC-theta**.

PKC-alpha, PKC-beta, PKC-gamma, and **PKC-epsilon** phosphorylate and activate c- **Raf-1**, thereby amplifying H-RAS/ MEK1 and MEK2/ ERK1/2 kinase cascade. **PKC-theta** activates Nuclear factor NF-kappa-B inhibitor kinase beta (IKK-beta) resulting in activation of the Nuclear factor NF-kappa-B (**NF-kB**).

The cytoplasmic tyrosine kinase **c-Src** is involved in important cellular processes such as mitogenic signaling or cytoskeletal organization. Substrates of the EGF-stimulated **c-Src** include the **EGFR** itself, transcription factors of the Signal transducer and activator of transcription family, such as **STAT3**, **Shc**, cytoskeletal components and some other proteins.

6. PDGF signaling via STATs and NF-kB

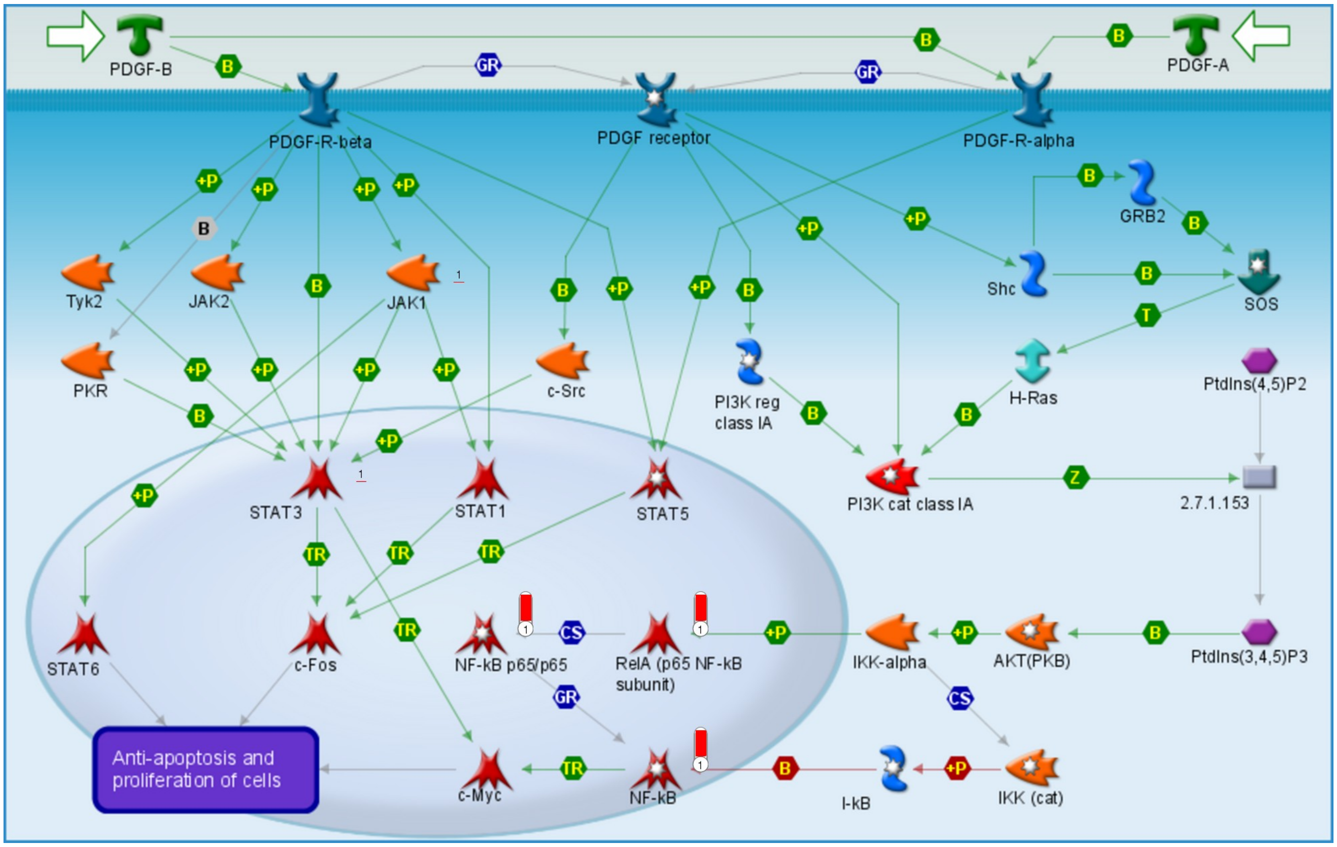

PDGF-induced anti-apoptosis and proliferation of cells via STAT and NF-KB pathways

Platelet-derived growth factors (**PDGF** s) are members of a large family of growth factors secreted by human vascular endothelial cells and fibroblasts.

The **PDGF** family is composed of four different polypeptide chains encoded by four different genes. There are two classical **PDGF** chains, **PDGF-A** and **PDGF-B**, and two only recently discovered chains, PDGF-C and PDGF-D. The four **PDGF** chains assemble into disulphide-bonded dimers via homo- or heterodimerization.

PDGF s regulate biological functions in cells through binding to specific structurally related high-affinity receptors (**PDGFR**) on cell surface, denoted PDGFR alpha and beta. Upon ligand binding, the **PDGFR** dimerizes and autophosphorylates on a number of tyrosine residues. Tyrosine phosphorylated

sites are used by **PDGFR** as anchor sites for various SH2 domain-containing proteins.

PDGF is a principal survival factor that inhibits apoptosis and promotes proliferation. The mechanisms of cellular proliferation and transformation are intrinsically linked to the process of apoptosis: the default of proliferating cells is to undergo apoptosis unless specific survival signals are provided.

It is show, that **PDGF-B** and sometimes **PDGF-A** regulate of cell growth and survival via the Signal transducer and activator of transcription (**STAT**) pathway and/or of through Nuclear factors of kappa light polypeptide in B-cells (**NF-KB)**. **PDGFR** -beta and, to a lesser degree, **PDGFR** -alpha participate in these processes.

Activated **PDGFR** s directly or indirectly (via activate members of the Janus kinase family (JAK) including **JAK1/JAK2** and/or Tyrosine kinase 2 (**TYK2**). **JAK1/JAK2** or **TYK2** signaling then lead to the stimulation of members of the STAT family (**STAT1**, **STAT3**, **STAT5**, **STAT6**). However, **STAT3** may also be stimulated by the proto-oncogene tyrosine-protein kinase (**c-Src**) and the Double-stranded RNA-activated protein kinase (**PKR**). **PKR** is pre-associated with **STAT3** and **PDGFR** -beta. It may facilitate tyrosine phosphorylation of **STAT3** by **c-Src**.

Activated **STAT** s participate in the survival and development of cells by regulating the expression of several genes such as proto-oncogene proteins **c-Fos** and **c-Myc**.

Upon **PDGF** stimulation, **PDGFRs** activate Phosphatidylinositol 3-kinase (**PI3K**) directly or indirectly (via Src homology 2 domain containing transforming protein (**Shc**)/ Factor receptor bound 2 (**Grb2**). The **PI3K** regulatory subunit (**PI3K reg 1A**) stimulates activity of **PI3K** catalytic subunits (**PI3K cat 1A**), which in turn catalyzes of reaction conversion Phosphatidylinositol-4,5-biphosphate (**PtdIns(4,5)P2**) into Phosphatidylinositol-3,4,5-trisphosphate (**PtdIns(3,4,5)P3**). **PtdIns(3,4,5)P3** binds to the pleckstrin-homology domain of serine/threonine protein kinase **Akt**, to recruit **Akt** to the plasma membrane. When **Akt** transiently associates with Inhibitor of nuclear factor kappa B kinase catalytic subunits (**IKK**), it phosphorilates and activates **IKK**. **IKK** phosphorylates and markes for degradation of NF-KB inhibitor (**I-KB**), thereby inducing **NF-kB** DNA-binding activity.

However, under certain circumstances, **Akt** can activate **NF-KB** through a mechanism that does not involve **I-KB** degradation by modulating the transcriptional potential of transcription factor p65 (**RelA**). **RelA** is a component of **NF-KB** complex.

NF-KB regulates transcription of **c-Myc**. **c-Myc** is a central regulator of cell growth, death and differentiation. **c-Myc** is required for cell proliferation but, in the absence of survival factors, it induces apoptosis. Thus, **PDGF** stimulates **c-Myc** -mediated proliferation by activating the **H-Ras/ PI3K/ Akt** pathway.

7. IGF_RI signaling

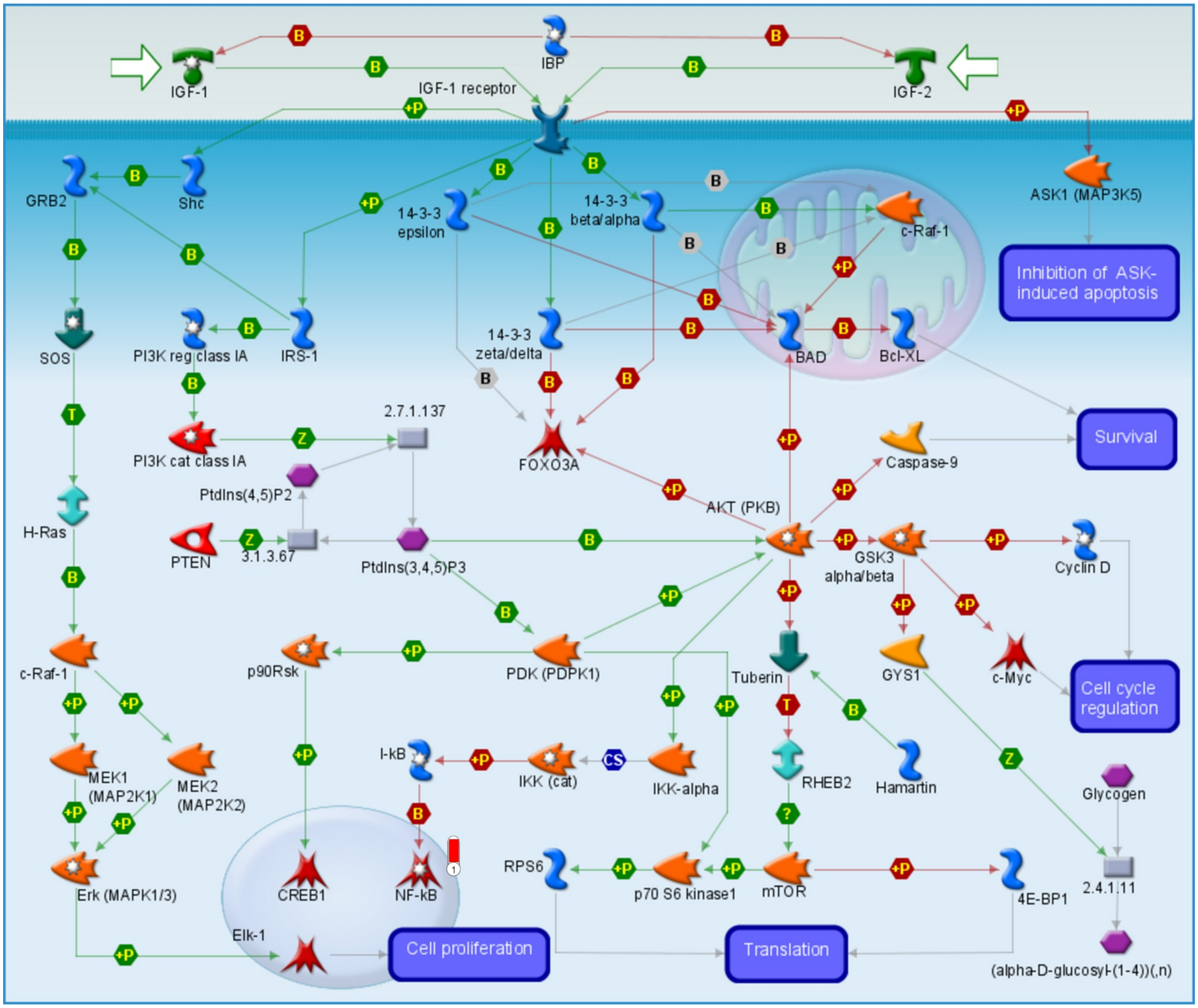

IGF-R1 signaling

The insulin-like growth factor system (IGF system) comprises two receptors: Insulin-like growth factor 1 receptor (**IGF-1 receptor**) and IGF-IIR with their respective ligands: Insulin-like growth factors 1 and 2 (**IGF-1** and **IGF-2**) and six high-affinity IGF binding proteins (**IBP**).

The principal processes mediated by the IGF system include stimulation of somatic growth by promoting cellular proliferation and differentiation. Additionally, it was shown that signaling through the **IGF-1 receptor** plays a critical role in cell survival and prevention of programmed cell death. In contrast, the IGF-IIR does not appear to be involved in the regulation of apoptosis.

Both **IGF-1** and **IGF-2** exhibit the high-affinity binding to **IGF-1 receptor**. The IGF binding proteins (**IBP**) bind to both **IGF-1** and **IGF-2** with high-affinity. Their main role is to modulate actions of free **IGF-1** and **IGF-2**.

IGF-1 receptor is a transmembrane tyrosine kinase receptor that is highly homologous to the insulin receptor (IR). Like IR, **IGF-1 receptor** consists of a2b2 heterotetramers held together by disulfide bridges. **IGF-1 receptor** and IR can also form heterodimers.

Binding of **IGF-1** and **IGF-2** to the cognate **IGF-1 receptor** stimulates the intrinsic tyrosine kinase activity of this receptor.

Upon **IGF** binding, the tyrosine kinase activity of **IGF-1 receptor** leads to the phosphorylation of several substrates, including the insulin receptor substrate family of proteins (such as Insulin receptor substrate 1 (**IRS-1**)), SHC (Src homology 2 domain containing) transforming protein 1 (**Shc**) and some others.

Once phosphorylated, these docking proteins activate downstream intracellular signaling through the Phosphatidylinositol 3-kinase (**PI3K**) or Growth factor receptor-bound protein 2 (**GRB2**)/ Son of sevenless homolog (**SOS**)/ v-Ha-ras Harvey rat sarcoma viral oncogene homolog (**H-Ras**) pathways that ultimately leads to cellular proliferation.

Activation of **IGF-1 receptor** by its ligand also initiates metabolic cascades that result in the stimulation of protein synthesis via activation of Ribosomal protein S6 kinase, 70kDa, polypeptide 1 (**p70 S6 kinase 1**), glucose uptake, glycogen synthesis, and lipid storage.

As mentioned above, **IGF-1** and **IGF-2** exhibit strong anti-apoptotic activity. There are three **IGF-1 receptor** -induced anti-apoptotic pathways. The main pathway for the antiapoptotic effect stimulated by **IGF-1 receptor** is the well-established **IRS-1** -mediated pathway that causes activation of **PI3K** and V-akt murine thymoma viral oncogene homolog 1 (**AKT(PKB)**), that leads to the phosphorylation of BCL2-associated agonist of cell death (**BAD**).

BAD is known to be a heterodimeric partner for both BCL2-like 1 (**Bcl-XL**) and B-cell CLL/lymphoma 2 (**Bcl-2**). BAD neutralizes **Bcl-XL** and **Bcl-2** protective effect and promotes cell death.

In its phosphorylated form, **BAD** is sequestered in the cytosol by **14-3-3** proteins and cannot bind to antiapoptotic proteins of the Bcl-2 family and therefore cannot induce cell death.

Another known anti-apoptotic pathway is mediated by **14-3-3** proteins.

Three members of the **14-3-3** family of proteins (Tyrosine 3-monooxygenase/tryptophan 5-monooxygenase activation protein, beta, zeta and epsilon polypeptides (**14-3-3 beta/alpha**, **14-3-3 zeta/delta**, and **14-3-3 epsilon**) interact with the **IGF-1 receptor**, after its autophosphorilation, in a variety of cultured cell types.

The **14-3-3** proteins have been implicated in the activation of v-raf-1 murine leukemia viral oncogene homolog 1 (**c-Raf-1**).

IGF-1 signaling leads to activation of **c-Raf-1** to promote its translocation to the mitochondria, where mitochondrial **c-Raf-1** phosphorylates **BAD**, causing its dissociation from antiapoptotic proteins (such as **Bcl-2** and **Bcl-XL**) and its release into the cytosol.

Additionally, **IGF-1 receptor** signaling suppresses the Mitogen-activated protein kinase kinase kinase 5 (**ASK1 (MAP3K5)**)-mediated stimulation of JNK/p38 and the induction of programmed cell death. **ASK1 (MAP3K5)** forms a complex with **IGF-1 receptor**. **IGF-1 receptor** specifically phosphorylates and inhibits **ASK1 (MAP3K5)**.

IRS proteins, including IRS -3 and IRS-4 however have a negative effect on the anti-apoptotic effects of **IGF-1**

8. AKT signaling

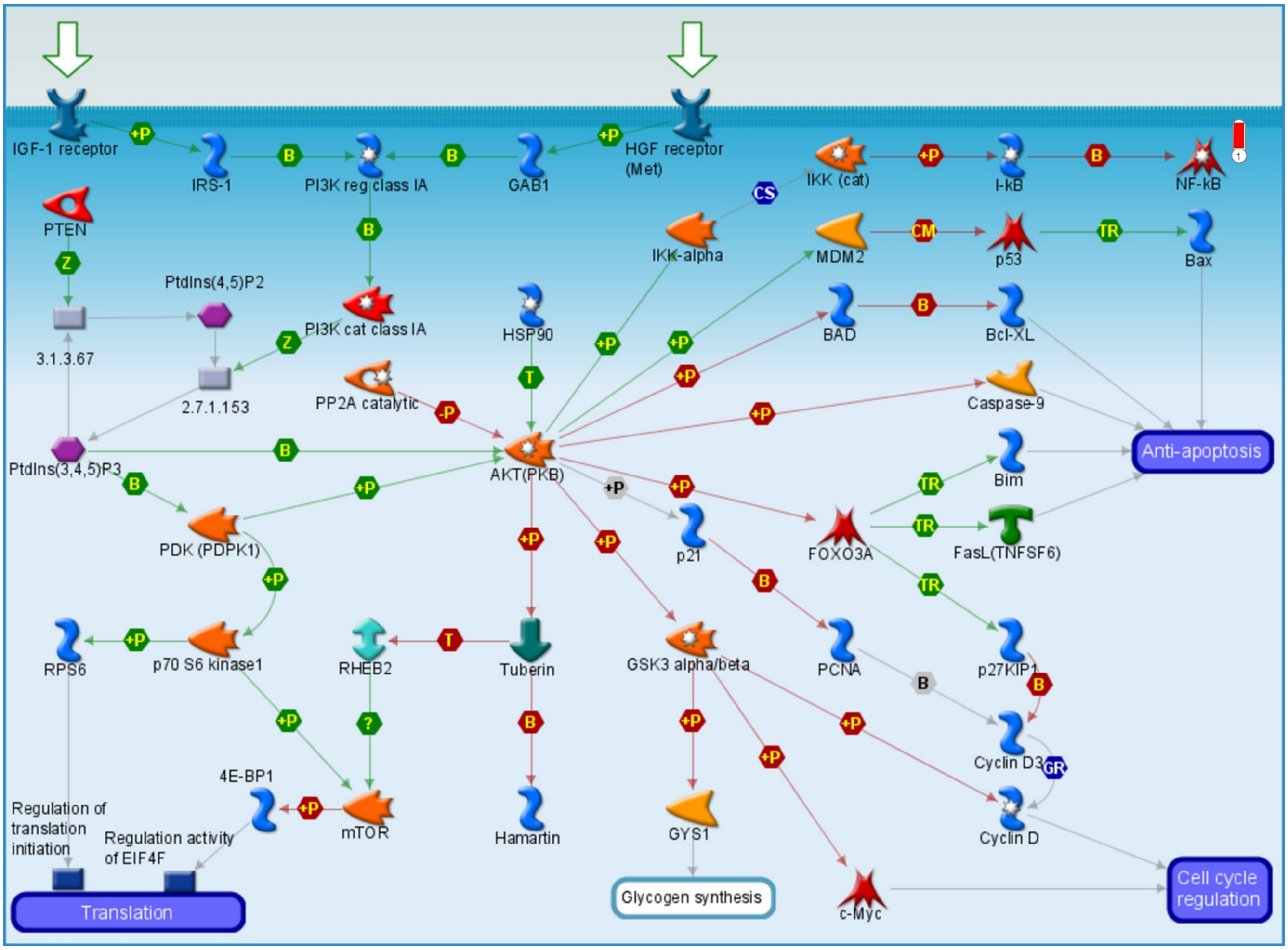

AKT signaling

RAC-alpha serine/threonine kinases (**AKTs**) are crucial mediators of various cellular process, such as apoptosis, regulation of cell cycle, protein synthesis and regulation of metabolism. The activity of **AKT** is modulated by various proteins, including Phosphatidylinositol-3-kinase (**PI3K**), Phosphoinositide-dependent kinase 1 (**PDK**), phosphotases **PTEN**, **PP2A**, and heat-shock protein (**Hsp90).**

PI3K converts phosphatidylinositol 4,5-biphosphate (**PI(4,5)P** $_2$) to phosphatidylinositol 3,4,5-triphosphate (**PI(3,4,5)P** $_3$), which is secondary messenger involved the in regulation of various process. **PI(3,4,5)P** $_3$ associates with the inner lipid bilayer of the plasma membrane and promotesthe recruitment of proteins with pleckstrin homology (PH) domains such as **AKT** and **PDK**. Upon binding to the membrane **AKT** and **PDK** became active. Notably, translocation of **AKT** to the plasma membrane also facilitates its phosphorylation by **PDK**.

AKT activity can be inhibited indirectly though the t phosphatase **PTEN** that cleaves the 3' phosphate from **PI(3,4,5)P** $_3$ to generate **PI(4,5)P** $_2$. **PTEN** therefore, acts to decrease levels of **PI(3,4,5)P** $_3$ causing an antagonistic effect of **AKT** -inducedcell survival.

The phosphatase **PP2A** dephosphorylates and inhibits **AKT** directly and this is counteracted by **Hsp90.**

Hsp90 forms a complex with **AKT** and prevents **PP2A** -mediated dephosphorylation. **Hsp90** plays an important role in maintaining **AKT** kinase activity.

Activated **AKT** prevents cells from undergoing apoptosis by inhibiting proapoptotic proteins BCL2-associated agonist of cell death (**BAD**) and **Caspase-9**. **AKT** induces phosphorylation of **BAD,** preventing **BAD** from binding with anti-apoptotic factor BCL2-like 1 (**BCL-X**) therby reducing antiapoptotic events.

AKT can interfere with cell death via a member of the forkhead family of transcription factors (e.g., Forkhead box O3 (**FOXO3A)**, which is a direct target for phosphorylation by **AKT)**. **FOXO3A** has been implicated in the expression of the FAS ligand (**FasL**) and the Bcl-2 interacting mediator of cell death (**Bim**), which can induce cell death. Upon phosphorylation by **AKT**, **FOXO3A** is retained in the cytosol preventing transcriptional regulation and expression of **FasL** and **Bim** in the nuclease, allowing the cell survival.

AKT also regulates the activity of other transcription factors, such as nuclear factor-kappaB (**NF-kB**), Tumor protein p53 (**p53**), c-Myc. **AKT** phosphorilates and activates I-kB kinase (**IKK**), that regulate the activity of the **NF-kB** transcription factor. When bound to its cytosolic inhibitor **I-kB**, **NF-kB** is inactive. Upon phosphorylation of **I-kB** by **IKK**, the inhibitor is degraded, allowing **NF-kB** to move to the nucleus and activate the transcription of antiapoptotic proteins.

AKT phosphorylates ubiquitin-protein ligase E3 Mdm2 p53 binding protein homolog (**MDM2**) that results in its translocation into the nucleus where it binds to transcription factor **p53**. **p53** mediates apoptosis through transactivation of apoptotic activator **BAX**. **MDM2** interacts with the **p53,** inhibits its transcriptional activity and targets it for degradation by the proteasome.

AKT affects the cell cycle progression by regulating the **Cyclin D** function. This is accomplished by phosphorylation of Cyclin-dependent kinase inhibitor 1A (**p21/WAF1)** by **AKT.** This result to cytoplasmic localization of **p21/WAF1**, thereby preventing its function in the nucleus. In the nucleous, the protein **p21/WAF1** interacts with and inhibits the essential DNA replication factor, proliferating-cell nuclear antigen (**PCNA**). **p21/WAF1** and **PCNA** forms complex with **Cyclin D**.

Another target for **AKT** is Glycogen synthase kinase 3 (**GSK3**), which negativly regulates glycogen synthesis and cell cycle progression via inhibitory phosphorylation of glycogen synthase and transcription factors **c-Myc** and **Cyclin D**, respectively.

Additionally, **FOXO3A** has been implicated in expression of the C yclin-dependent kinase inhibitor 1B (**P27KIP1**), which binds with and inhibits **Cyclin D**.

The activation of **AKT** results in the stimulation of protein synthesis via activation of Ribosomal protein S6 kinase (**p70S6K**). The activation of **p70S6K** by **AKT** occurs via direct and indirect mechanisms. The indirect process is mediated by FKBP-rapamycin associated protein (**FRAP1**). In absence of **AKT** -mediated phosphorylation, **Tuberin** via the small GTPase **Rheb** inhibits **FRAP1**, allowing the activation of **P70S6K** the leads to multiple phosphorylation events of 40S ribosomal protein S6 (**RPS6**) to trigger protein synthesis.

9. TGF, WNT and cytoskeletal remodeling

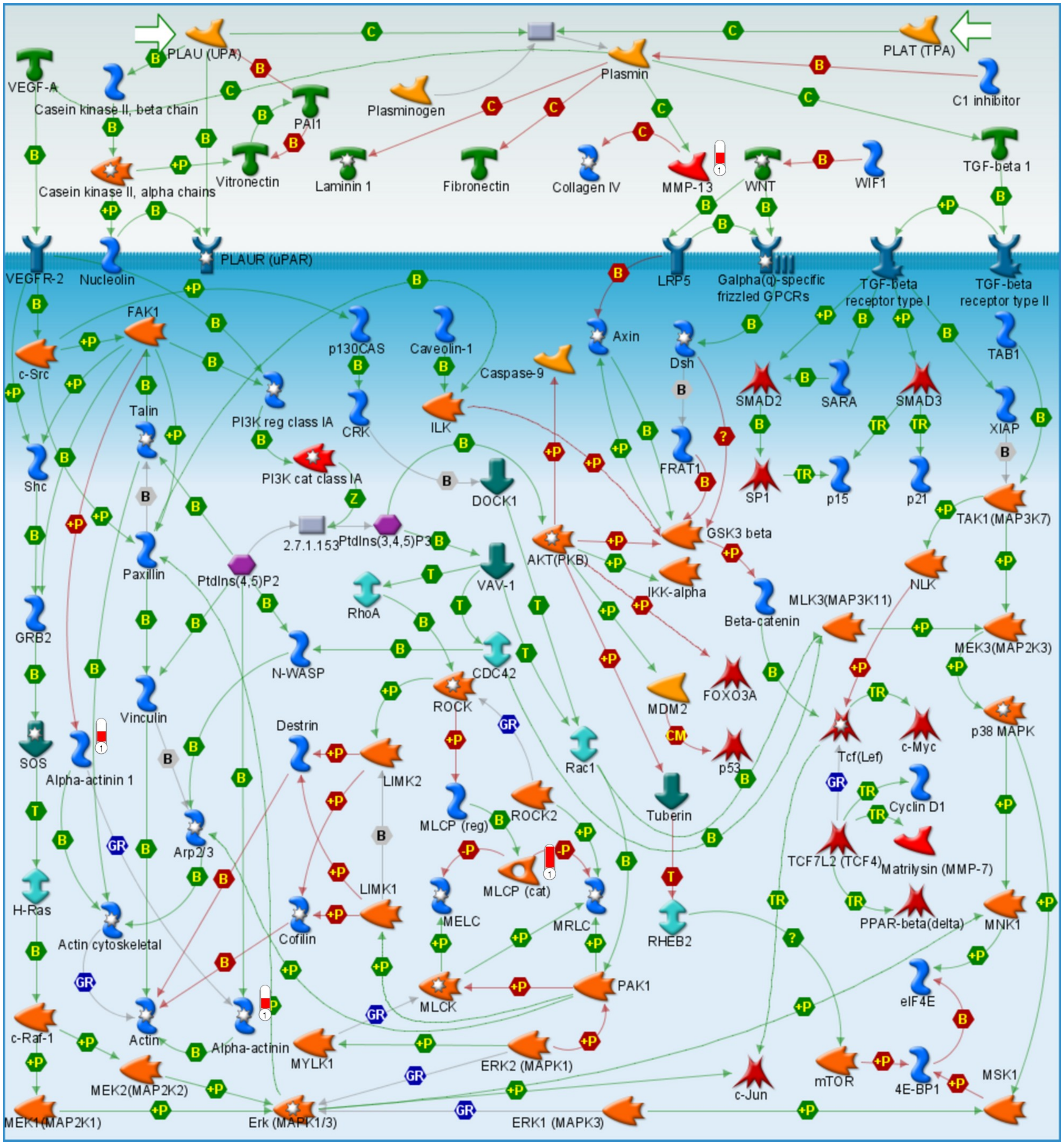

II. Pub-med hits and functional analysis of downregulated genes in psoriasis

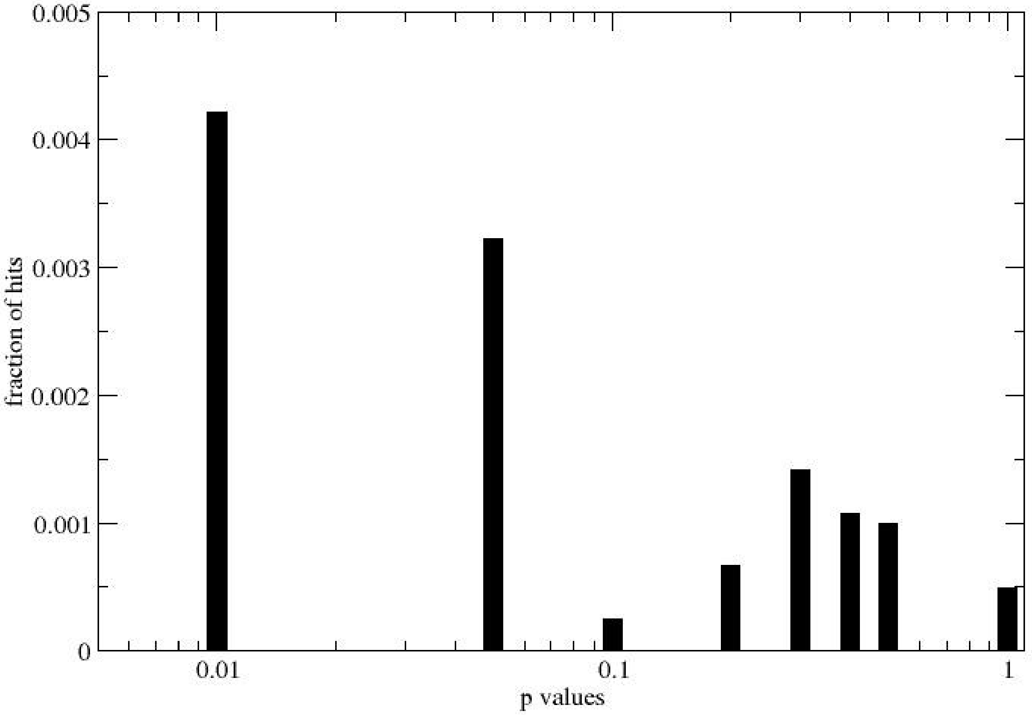

(1) Fraction of pubmed hits of genes from the shortest paths connecting the set of downregulated genes in psoriasis

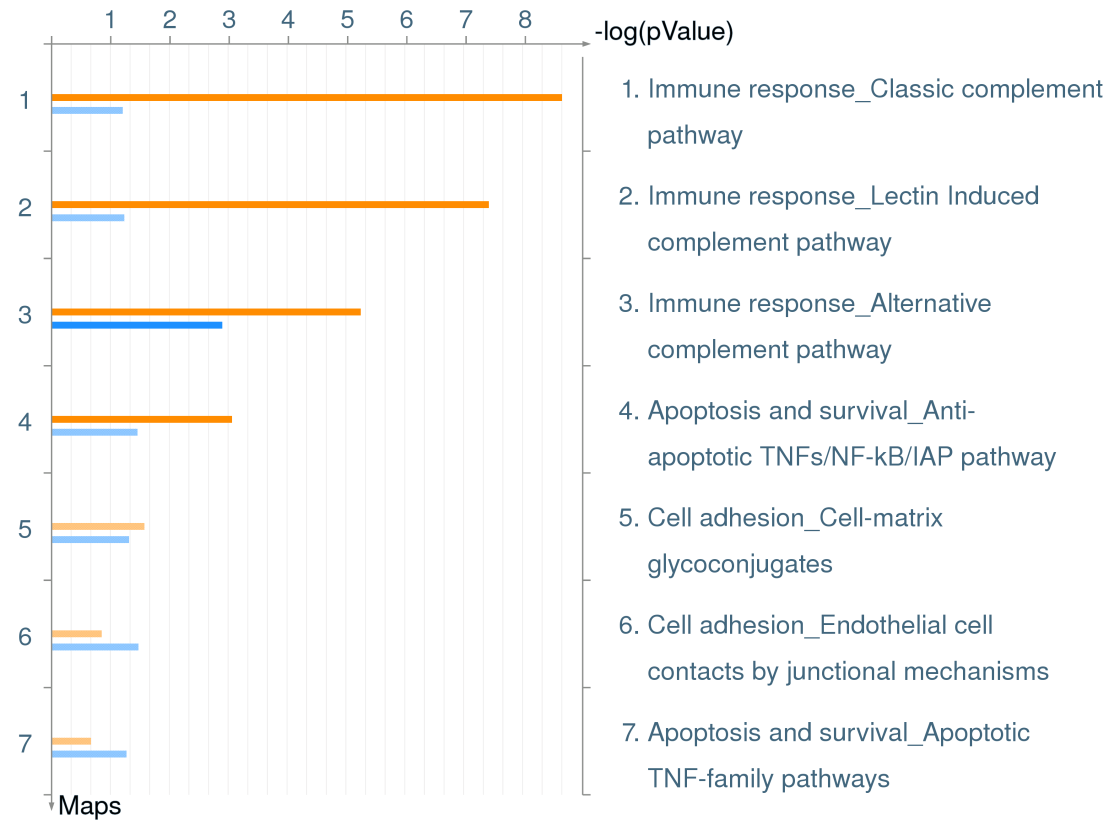

(2) Functional analysis of the topologically significant genes connecting the set of downregulated genes in psoriasis

III. **Pub-Med hits of genes with different diseases**

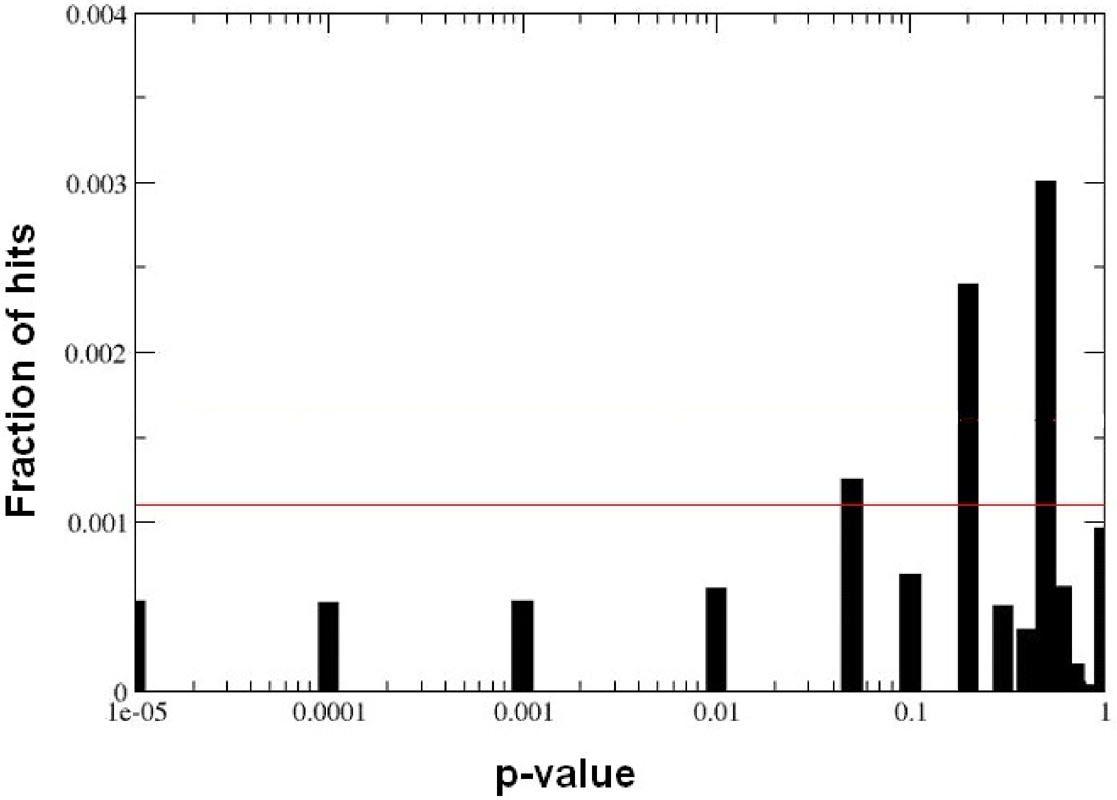

(1) Co-occurrence of topologcally significant gene names with the word “glaucoma” as a fraction of total number of PubMed hits. Here we verify that the high scored nodes for psoriasis do not positively correlate with the number of literature hits for some unrelated disease. The fractions of Pubmed hits does not depend on the p values (almost constant), the high fluctuation at larger p values is due to the decrease in the averaged sample size. Also, the fraction of hits for low p values does not exceed of that of a random set of genes of the same size (red line).

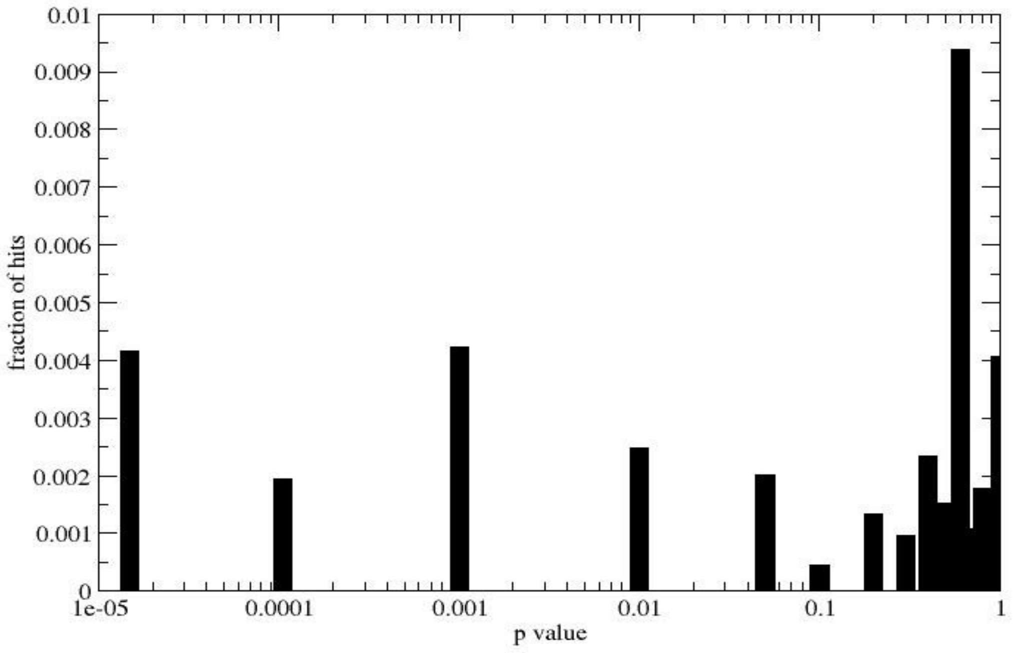

(2) The fraction of pub-med hits with “multiple sclerosis”, an autoimmune disease. The scaling of the PubMed hits was not as good as it was in the case of “psoriasis”, but for low p values the fraction of hits was still slightly higher. This is expected because both diseases are autoimmune and therefore they probably share some of the affected pathways.
