# Supplementary material for: Identifying disease-specific genes based on their topological significance in protein networks
Source: BMC Syst Biol. 2009 Mar 23;3:36. doi: 10.1186/1752-0509-3-36 (PMC2678983; doi:10.1186/1752-0509-3-36)
Supplement: Additional file 7 — Enrichment analysis of functional hubs. We built network modules consisting of sets of genes linked to functional hubs by upstream or downstream functional connections. Functional enrichment analysis was performed to prioritize hubs based on their relevance to psoriasis-related processes or psoriasis-related genes. [file 1752-0509-3-36-S7.pdf]

**Convergence hubs**

| | | | |
|---|---|---|---|
| MGMT | Skin Diseases | Breast Neoplasms | Breast Diseases |
| | Skin and Connective Tissue Diseases | Carcinoma | |
| ITIH4 | Neoplasms | Neoplasms by Site | Urogenital Neoplasms |
| | Urogenital Diseases | Skin and Connective Tissue Diseases | |
| RAR-alpha/RXR-alpha | Skin Diseases | Digestive System Diseases | Gastrointestinal Neoplasms |
| | Digestive System Neoplasms | Skin and Connective Tissue Diseases | |
| Coagulation factor XII | Skin Diseases | Skin and Connective Tissue Diseases | Digestive System Neoplasms |
| | Neoplasms by Site | Breast Neoplasms | |
| YY1 | Neoplasms by Site | Digestive System Neoplasms | Urogenital Neoplasms |
| | Digestive System Diseases | Breast Neoplasms | |
| NFKBIA | Virus Diseases | Digestive System Diseases | Hemic and Lymphatic Diseases |
| | Intestinal Diseases | Skin Diseases | |
| KNG | Pathologic Processes | Skin and Connective Tissue Diseases | Digestive System Diseases |
| | Urogenital Diseases | Female Genital Diseases and Pregnancy Complications | |
| B2 bradykinin receptor | Digestive System Neoplasms | Skin Diseases | Gastrointestinal Neoplasms |
| | Digestive System Diseases | Skin and Connective Tissue Diseases | |
| EMAPII | Virus Diseases | Digestive System Diseases | |
| | Gastrointestinal Diseases | Carcinoma | Intestinal Diseases |
| HNF4-alpha | Genital Neoplasms, Male | Prostatic Neoplasms | Prostatic Diseases |
| | Genital Diseases, Male | Hepatitis B | |
| 26S proteasome (20S core) | Neoplasms | Skin Diseases | Neoplasms by Site |
| | Skin and Connective Tissue Diseases | Digestive System Diseases | |
| NF-kB1 (p105) | Skin and Connective Tissue Diseases | Skin Diseases | Urogenital Diseases |
| | Urogenital Neoplasms | Genital Diseases, Female | |
| p53 | Gastrointestinal Neoplasms | Digestive System Neoplasms | Gastrointestinal Diseases |
| | Colorectal Neoplasms | Rectal Diseases | |
| TRPA1 | Signs and Symptoms, Respiratory | Respiratory Tract Diseases | Pathologic Processes |
| | Lung Diseases | Urogenital Diseases | |
| Thrombospondin 1 | Pathologic Processes | Pathological Conditions, Signs and Symptoms | Skin Diseases |
| | Skin and Connective Tissue Diseases | Breast Neoplasms | |

| | | | |
|---|---|---|---|
| ROR-alpha | Skin Diseases | Breast Neoplasms | Breast Diseases |
| | Skin and Connective Tissue Diseases | Neoplasm Metastasis | |
| Trypsin | Urogenital Diseases | Female Genital Diseases and Pregnancy Complications | Urogenital Neoplasms |
| | Skin Diseases | Skin and Connective Tissue Diseases | |
| NFIC | Pathologic Processes | Pathological Conditions, Signs and Symptoms | Carcinoma, Renal Cell |
| | Pancreatic Diseases | Embryo Loss | |
| IL-1 beta | Pathologic Processes | Skin Diseases | Skin and Connective Tissue Diseases |
| | Pathological Conditions, Signs and Symptoms | Digestive System Diseases | |
| Cathepsin G | Neoplasms | Skin and Connective Tissue Diseases | Neoplasms by Site |
| | Skin Diseases | Neoplasms by Histologic Type | |

**Divergence hubs**

| | | | |
|---|---|---|---|
| IL-6 | Inappropriate ADH Syndrome | Hypopigmentation | Pancreatitis |
| | Retinal Artery Occlusion | Craniopharyngioma | |
| IRF8 | Adenocarcinoma | illness | Carcinoma |
| | Neoplasms by Site | Digestive System Neoplasms | |
| IRF2 | Genital Neoplasms, Female | Adnexal Diseases | Epstein-Barr Virus Infections |
| | Neoplasm Metastasis | Ovarian Neoplasms | |
| STAT1 | Skin Diseases | Neoplasms by Site | Skin and Connective Tissue Diseases |
| | Breast Neoplasms | Breast Diseases | |
| IFN-gamma | Skin Diseases | Skin and Connective Tissue Diseases | Breast Neoplasms |
| | Breast Diseases | Adnexal Diseases | |
| PSMA2 | Carcinoma | Carcinoma, Small Cell | Carcinoma, Neuroendocrine |
| | Neoplasms, Glandular and Epithelial | Carcinoma, Squamous Cell | |
| TNF-alpha | Tuberous Sclerosis | Cardiomegaly | Hamartoma |
| | Schizophrenia, Paranoid | Schizophrenia | |
| IRF9 | Pancreatitis | Lipodystrophy | Digestive System Diseases |
| | Pancreatic Diseases | Multiple Sclerosis | |
| STAT2 | Digestive System Diseases | Dermatitis, Atopic | Skin Diseases, Eczematous |
| | Dermatitis | Carcinoma, Ductal, Breast | |
| c-Jun | Alcoholic Neuropathy | Skin Diseases | Flushing |
| | Menkes Kinky Hair Syndrome | Colorectal Neoplasms | |

| | | | |
|---|---|---|---|
| IRF1 | Digestive System Diseases | Digestive System Neoplasms | Neoplasms |
| | Skin Diseases | Hemorrhagic Fevers, Viral | |

Is drug target

Is drug target and related to skin diseases

Related to skin diseases, not a known target
